# Two Complementary Signaling Pathways Depict Eukaryotic Chemotaxis: A Mechanochemical Coupling Model

**DOI:** 10.3389/fcell.2021.786254

**Published:** 2021-11-17

**Authors:** Lüwen Zhou, Shiliang Feng, Long Li, Shouqin Lü, Yan Zhang, Mian Long

**Affiliations:** ^1^ Smart Materials and Advanced Structure Laboratory, School of Mechanical Engineering and Mechanics, Ningbo University, Ningbo Zhejiang, China; ^2^ Key Laboratory of Microgravity (National Microgravity Laboratory), and Beijing Key Laboratory of Engineered Construction and Mechanobiology, Center for Biomechanics and Bioengineering, Institute of Mechanics, Chinese Academy of Sciences, Beijing, China; ^3^ State Key Laboratory of Nonlinear Mechanics (LNM) and Beijing Key Laboratory of Engineered Construction and Mechanobiology, Institute of Mechanics, Chinese Academy of Sciences, Beijing, China; ^4^ School of Engineering Science, University of Chinese Academy of Sciences, Beijing, China

**Keywords:** chemotaxis, cytoskeletal remodeling, mathematical model, biochemical, biomechanical

## Abstract

Many eukaryotic cells, including neutrophils and *Dictyostelium* cells, are able to undergo correlated random migration in the absence of directional cues while reacting to shallow gradients of chemoattractants with exquisite precision. Although progress has been made with regard to molecular identities, it remains elusive how molecular mechanics are integrated with cell mechanics to initiate and manipulate cell motility. Here, we propose a two dimensional (2D) cell migration model wherein a multilayered dynamic seesaw mechanism is accompanied by a mechanical strain-based inhibition mechanism. In biology, these two mechanisms can be mapped onto the biochemical feedback between phosphoinositides (PIs) and Rho GTPase and the mechanical interplay between filamin A (FLNa) and FilGAP. Cell migration and the accompanying morphological changes are demonstrated in numerical simulations using a particle-spring model, and the diffusion in the cell membrane are simulations using a one dimensional (1D) finite differences method (FDM). The fine balance established between endogenous signaling and a mechanically governed inactivation scheme ensures the endogenous cycle of self-organizing pseudopods, accounting for the correlated random migration. Furthermore, this model cell manifests directional and adaptable responses to shallow graded signaling, depending on the overwhelming effect of the graded stimuli guidance on strain-based inhibition. Finally, the model cell becomes trapped within an obstacle-ridden spatial region, manifesting a shuttle run for local explorations and can chemotactically “escape”, illustrating again the balance required in the complementary signaling pathways.

## 1 Introduction

Chemotaxis is characterized by the directed movement of cells along a chemotactic gradient and is crucial for many biological processes, including neuronal patterning (Castellani, 2013), wound healing (Andasari et al., 2018), immune response (Kolaczkowska and Kubes, 2013), and cancer metastasis (Stephens et al., 2008; Polacheck et al., 2013). Even apart from its significant biological function, chemotaxis is an invaluable system for understanding intracellular signaling cascades. For cells to achieve effective movement in response to extracellular guidance, their intracellular signaling network responsible for cytoskeleton remodeling must be differentially and persistently regulated (Petrie et al., 2009; Welf and Haugh, 2011). Studies using modeled organisms, such as neutrophils and *Dictyostelium* cells, have shown that the molecular mechanisms of chemotaxis are largely shared (Parent, 2004; Van Haastert and Devreotes, 2004), indicating an underlying intrinsic building strategy in physics.

To our knowledge, the spatiotemporal regulation of the biochemical network responsible for eukaryotic chemotaxis can be understood within a dynamic seesaw modeling framework wherein a series of “symmetry breaking” events are involved in different signaling layers (Weiner et al., 2002). At the top layer, cells use uniformly distributed G-protein coupled receptors (GPCRs) to sense the concentration field of a chemoattractant; then, the associated G protein dissociates into G_α_ and G_βγ_ subunits, which trigger a fast activation response and a slow inhibition response to achieve directional sensing (Janetopoulos et al., 2004; Levine et al., 2006; Gambardella and Vermeren, 2013). In the middle layer, Rho GTPases (Cdc42, Rac, RhoA) serve as central hubs in transducing signals from the extracellular space to the cytoskeleton (Fukata et al., 2003; Raftopoulou and Hall, 2004). These Rho GTPases are switch-like proteins that are cycled between active membrane-bound (GTP) forms and inactive cytosolic (GDP) forms (Raftopoulou and Hall, 2004), and the existing mutual antagonism effects ensure that the same external signal may determine the features of both the front and the rear of the cell (Onsum and Rao, 2005). At the bottom layer, active Rho GTPases, together with the regulation of phosphatidylinositol-3-kinase (PI3K) and phosphatidylinositol phosphatase (PTEN) (Comer and Parent, 2002; Wang et al., 2002; Billadeau, 2008), contribute to the asymmetric phosphatidylinositol 3, 4, 5-trisphosphate (PIP_3_) distribution by forming positive/negative feedback loops (Weiner et al., 2002).

Once the spatial effect of such feedback loops has been fully evoked, the cell acts as a seesaw that persists to amplify signaling in a bidirectional manner until an “all-or-none” distribution pattern of molecular components has been achieved (Weiner et al., 2002). For a well-polarized cell, the region of high Cdc42/Rac activity and the activation of Arp2/3 promotes actin branching, thus forming a protrusive pseudopod (Francakoh and Devreotes, 2004; Van Haastert P. J. M., 2010); the region of high RhoA activity and the activation of Rho kinase (ROCK) phosphorylates myosin phosphatase, triggering tail retraction (Wong et al., 2006; Rottner and Stradal, 2011). While the above biochemical components and their interactions are well characterized, the importance of the biomechanical pathways, as represented by FLNa-FilGAP (Nakamura, 2013), is only beginning to be revealed. As the cell extends its pseudopods, FilGAP dissociates from FLNa in actin networks by the myosin-dependent mechanical deformation of the actin network (Nakamura, 2013). FilGAP then relocates to the plasma membrane, where it antagonizes Rac and thus F-actin polarity (Plotnikov et al., 2012). Such a strong dependence between mechanical stretching and FilGAP release has been further proven in reconstitution studies with purified actin cytoskeletal components (Ehrlicher et al., 2011).

A body of mathematical modeling work has been performed for eukaryotic chemotaxis (Devreotes and Janetopoulos, 2003; Danuser et al., 2013). A common feature of these models is the incorporation of diffusion-reaction systems, representing the development of cell polarity, together with a mechanical model of the cell cortex (Holmes and Edelsteinkeshet, 2012). One popular model is based on the level set method (LSM) (Yang et al., 2008). For example, a local excitation, global inhibition biased excitable network (LEGI-BEN) mechanism was incorporated to account for the chemotaxing behavior of cells (Shi et al., 2013). A wave-pinning (WP) mechanism for Rac/Rho signaling was applied to reveal how a cell regulates its shape (Wolgemuth et al., 2010). A layered signaling model was therein proposed to examine how cell shape influences biochemical repolarization in stable keratocyte motion. While these works are helpful as they offer fundamental insights into eukaryotic chemotaxis, the role of biomechanical pathways is largely unknown in those models in the features of chemotaxis.

In this work, we present a mathematical model for eukaryotic chemotaxis with complementary biochemical and biomechanical signaling pathways. We started by using our previous work (Feng et al., 2018), and extended it in two aspects. First, a FLNa-FilGAP pathway was incorporated in a discrete network of actin filaments. Second, the reaction-diffusion in cell membrane is reduced from 2D to 1D for more reasonable and faster computing speed. Our model attempts to incorporate the following experimental findings into an integrative modeling framework: how a cell 1) exhibits correlated random migration in the absence of a chemoattractant stimulus, 2) generates directional and adaptable migration in response to a shallow graded stimulus, and 3) migrates chemotactically within an obstacle-ridden spatial region.

## 2 Methods

**FIGURE 1:**
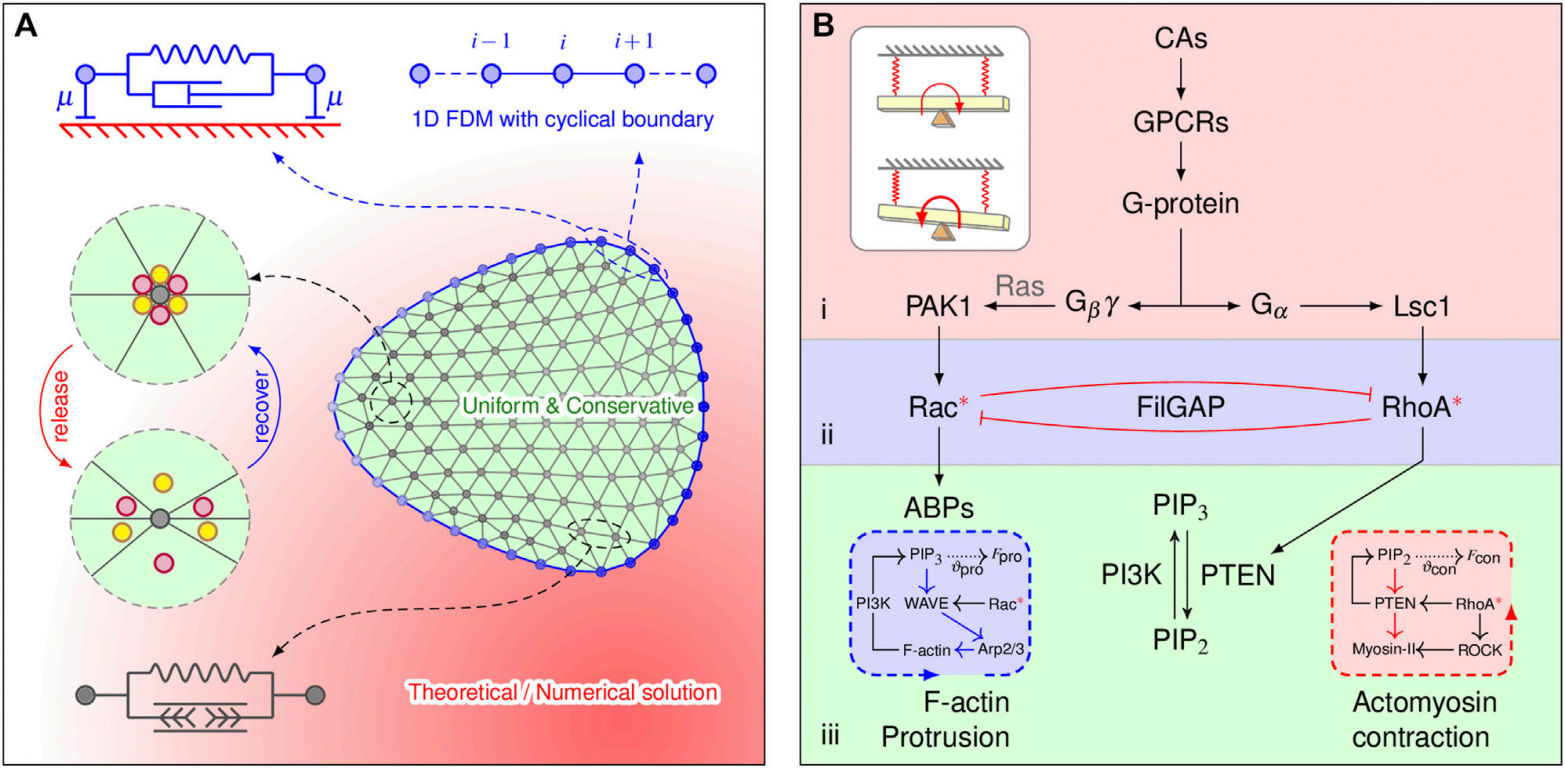
Model components and implications. **(A)** Two-dimensional cell mesh and the mechanical properties of the elements. The membrane element (blue) consists of a spring of stiffness *k* and a dashpot of viscosity *η* that are connected in parallel. The dashpot with a damping coefficient *μ* connected to the substrate accounts for the viscous dissipation. The lamellipodia are triangulated, and the lamellipodial element consists of an elastic element. FilGAP molecules, marked by colored balls, can either be released from or return to actin-FLNa crosslinking, depending on angular strain. Diffusion-reaction processes are explicitly considered only at the membrane element, implemented by the 1D FDM. **(B)** Multilayered signaling cascades responsible for cytoskeletal remodeling. 1) Initial signal processing: a cell detects chemoattractants (CAs) by G protein-coupled receptors (GPCRs); the G protein then dissociates into G_*α*_ and G_βγ_ subunits. This layer is closed if no chemotaxis signaling is applied. 2) Small Rho GTPase regulation: spatial regulation of Rac and RhoA by CA is achieved by the localization of GEFs (*i.e.*, PAK1 and Lsc1); releasing FilGAP may downregulate Rac and upregulate RhoA *via* their antagonistic effects. 3) Bidirectional molecular transport: spatial effects generated from Rho GTPase-PI feedback loops manipulate bidirectional cytoskeletal remodeling. Inserted blue and red boxes show the protrusive and contractive force generation mechanisms, respectively, which are inexplicitly considered. The inserted white plain box shows how such signaling cascades work in physics (a detailed explanation can be found in the discussion).

Our 2D model structurally comprises membrane actin and a discrete network of actin filaments (Figure 1A). What we called “the membrane” in the model represents two biological entities: the plasma membrane and the underlying cortex. The membranous viscoelastic element consists of a spring of stiffness *k* and a dashpot of viscosity *η* that are connected in parallel. The 2D lamellipod is modeled by *N* nodes connected by the edges of the Delaunay triangulation. Each lamellipodial element consists of a Hookean spring with stiffness *K* and a rest length of zero. Our cell migration model strives to capture not only the overall mechanics of cell deformation but also the intracellular signaling events. Therefore, our model can be organized into three modules: the cell mechanics module, biochemistry module, and mechano-sensing module. In the following subsections, we will discuss each of the three modules. As the nucleus and other cytoplasmic organelles play insignificant roles in the phenomenon of interest, they will not be explicitly modeled. Mathematical details and governing assumptions of the model are described in the Supplementary Material.

### 2.1 Cell Mechanics

The cell membrane is represented by 2D spring-connected particle circle Gallage et al. (2013) (Figure 1A). The motion of each node *i* on the cell membrane is described by the following force balance equation:

$$\mu v_{i}=F_{i}^{\text{elas}}+F_{i}^{\text{vis}}+F_{i}^{\text{pro}}+F_{i}^{\text{cont}}+F_{i}^{\text{drag}}$$ (1)

where **v**_*i*_ is the velocity vector, *μ* is the equivalent friction coefficient, and $F_{i}^{\text{elas}}$ , $F_{i}^{\text{vis}}$ , $F_{i}^{\text{pro}}$ , $F_{i}^{\text{cont}}$ and $F_{i}^{\text{drag}}$ are the elastic energy force, viscous energy force, protrusive force, contractive force, and drag force, respectively. Expressions for each term are provided in the Supplementary Material.

Cell membrane nodes provide the first boundary condition to lamellipodial nodes, a very good assumption according to the experimental observations that no slippage of the lamellipod is visualized at the leading edge (Paul et al., 2008). The position of the *i*th lamellipodial node is determined according to the force balance,

$$\underset{j}{\sum }\left(X_{j}-X_{i}\right)=0$$ (2)

where **X**_*j*_ and **X**_*i*_ are the position vector of the edge connecting the *i*th and *j*th nodes, and the summation includes all nodes connected to the given node by a Delaunay edge.

### 2.2 Biochemistry of the Model

The biochemical signaling pathways used in our model are summarized schematically in Figure 1B. Briefly, the model consists of a set of coupled partial differential equations (PDEs) that describe the kinetics, crosstalk, diffusion, and exchange of the signaling molecules and are assigned to three submodules: 1) initial signaling processing, 2) small Rho GTPase regulation, and 3) bidirectional molecular transport. The equations for modules 1) and 3) are in accordance with the balanced inactivation mechanism (Levine et al., 2006) and our previous work (Feng et al., 2018), respectively. The spatiotemporal regulations of active Rac/Rho are described by the following equations:

$$\frac{\partial G}{\partial t}=D_{m}\nabla^{2}G+P_{G}\left(R,\rho \right)G_{i}-\delta_{G}G+Q_{G}G,$$ (3)

where *G* = *R* and *ρ* represent the active forms (membrane-bound) of Rac and RhoA, respectively. *G*_*i*_ = *R*_*i*_, and *ρ*_*i*_ are the total amounts of the inactive forms of Rac and RhoA, respectively, which can be calculated directly by the conservation law. *P*_*G*_ is the activation term and is expressed as

$$P_{R}=I_{R}+\alpha E_{P}\;\text{and}\;P_{\rho }=I_{\rho }+\tau E_{\rho }.$$ (4)

Here, *I*_*R*_ and *I*_*ρ*_ are the respective baseline activation rates. *α* and *τ* are the activation rates of Rac and RhoA by PAK1 (*E*_*p*_) and Lsc1 (*E*_*ρ*_), respectively, and their concentrations are transmitted from submodule (i). *δ*_*G*_ is the GAP-mediated baseline inactivation rate. *Q*_*G*_ represents the FilGAP-mediated antagonism effect between Rac and RhoA and is determined by the first-order ultrasensitivity mechanism as follows (Lipshtat et al., 2010):

$$Q_{R}=-Q_{\rho }=-\frac{1}{1+A_{G}\exp\left(-b\left[\text{FilGAP}\right]\cdot \left[{\text{PIP}}_{3}\right]\right)}$$ (5)

Here, *A*_*G*_ determined the steepness of this transition, and the parameter *b* is a measure of the signal impact (Lipshtat et al., 2010).

### 2.3 Mechano-Sensing Mechanism

The release rate of FLNa-bound FilGAP is mediated by a strain-governed bandpass mechanism (Kang et al., 2015). That is, if an angle change Δ*θ*_*i*_ falls inside range [*β*_1_, *β*_2_], the FLNa crosslink is in a conformational state of slowly releasing FilGAP; otherwise, the crosslink is in a fast releasing state. The temporal evolution of the number of FilGAPs (*m*) remaining in a specific network can be described by

$$\begin{array}{cc} \frac{dm_{i}}{dt}= & A\cdot \left(m_{i}^{0}-m_{i}\right)\left(\underset{i}{\sum }m_{i}^{0}-\underset{i}{\sum }m_{i}\right)\\ & -\left[k_{\text{slow}}+\left(k_{\text{fast}}-k_{\text{slow}}\right)\cdot sng\left(\Delta \theta_{i},t\right)\right] \end{array}$$ (6)

where *A* is a constant and *k*_slow_ and *k*_fast_ are slow and fast release constants, respectively. Additionally, sng (Δ*θ*_*i*_, *t*) is an indicator function, which is used to switch between slow and fast releasing FilGAP states. The total amount of FilGAP remains conserved throughout the simulations, such that the concentration of cytosolic FilGAP can be derived from the conservation law.

### 2.4 Computational Setup and Numerical Methods

The cell diameter was set as 10 μm. The mesh parameters used were 421 internal nodes and 45 peripheral nodes for a cell. It is assumed that the cell is chemotactically responsive in a sufficiently large geometry (without boundary effect); otherwise, the first boundary condition is applied to confine cell movement in the presence of obstacles (see Supplementary Material). Since the cell shape and signaling concurrently influence each other, the arising mechano-chemical coupling problem is treated by 1D FDM according to Fick’s laws of diffusion (Merchant et al., 2018a). The diffusion of signaling molecules on the membrane is explicit, while the cytosolic components are assumed to be spatially uniform. A simple Monte-Carlo (MC) method was adopted to evaluate the translocation behaviors of effector molecules (*i.e.*, PI3K/PTEN). Detailed descriptions of the simulation procedures are presented in the Supplementary Material.

## 3 Results

### 3.1 Correlated Random Migration

**FIGURE 2:**
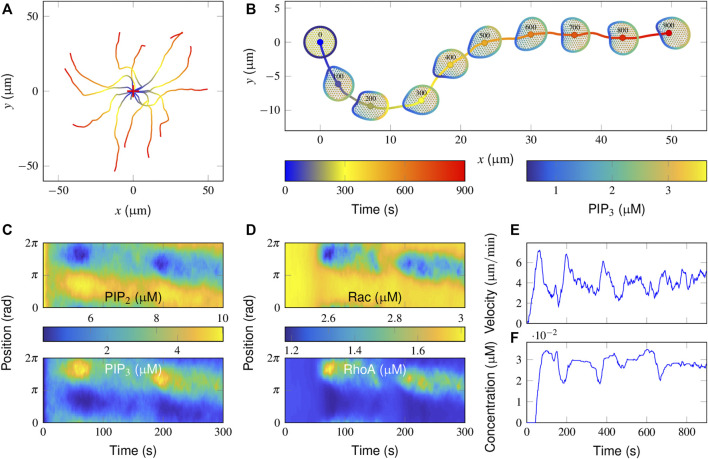
Simulation of correlated random migration in the absence of an attractant stimulus. **(A)** Cell centroid trajectories from 15 simulation runs of 900 s each. The cross symbol denotes the starting point. **(B)** Successive snapshots from a typical simulation. Colored plots are presented of the PIP_3_ concentrations on the cell membrane and that of FilGAP on the deformed FLNa network. **(C,D)** Spatiotemporal regulation of PIP_3_ **(C up)**, PIP_2_ **(C bottom)**, active Rac **(D up)**, and RhoA **(D bottom)** during the initial 300 s of the above typical simulation in **B**. **(E,F)** Temporal evolution of the cell movement velocity **(E)** and the cytosolic FilGAP concentration **(F)**.

To illustrate how a cell moves without chemoattractant guidance, we allowed the model cell to move in a simulation run for 900 s with default parameter values (Supplementary Table S1, S2) and repeated the simulations independently for 15 runs. Each simulation was initiated by placing the cell in the same position. The trajectories of cell centroids in each run were recorded and are presented in Figure 2A. The directions of cell migration are completely different among the repeated runs. The dynamics of how the local PIP_3_ concentration determines the direction of cell movement are presented in Figure 2B (also see Supplementary Video S1). Clearly, once moved, the cell takes a roughly oval-shaped morphology and exhibits a correlated random walk: the direction of future movement is correlated with that of prior movement, and the cell consequently moves with persistence.

To interpret the molecular mechanisms of such cellular behaviors, spatiotemporal plots of PIP_3_/PIP_2_ and active Rac/RhoA derived from this representative simulation are further demonstrated (Figures 2C,D). Note that the subsequent hotspots appeared at different positions in the PIP_3_ plot (Figure 2C up), suggesting that the cell generates different pseudopods. More specifically, the distribution of active Rac and RhoA remained uniform during the initial 50 s (Figure 2D). The baseline activities of Rac and RhoA provided a limited “driving force” for evoking cytoskeletal remodeling. In fact, PI3K and PTEN, acting as effectors, were able to translocate from the cytosol to the membrane *via* PIP_3_ and PIP_2_, respectively. After being activated by active Rac and RhoA, the catalytic effect of PI3K leads to the conversion of PIP_3_ and PIP_2_ and *vice versa* for PTEN.

Since membrane diffusion is small, a local increase in PIP_3_ concentration could be confined locally, resulting in further recruitment of PI3K. A patch of PIP_3_ is then formed during 30–75 s. As PIP_2_ has the same diffusivity as PIP_3_ and their total amounts are constant, the pattern of PIP_2_ regulation mirrors that of PIP_3_ (Figure 2C). The regions with high PIP_3_ and PIP_2_ are interpreted as the sites where protrusive and contractive force would be enhanced. The accompanying deformation of the actin network also results in the release of FilGAP from the FLNa crosslinks, such that the higher the degree of deformation is, the lower the concentration of FilGAP remains (Figure 2B). After binding with PIP_3_, FilGAP starts to downregulate the activity of Rac and upregulate that of RhoA, which together results in a local decrease in PIP_3_ (Figure 2B, 75–175 s). The adjacent regions receive the highest signal amplification and a moderate level of inhibition, defining a narrow range of locations where new pseudopods can be generated.

To further quantify cell motility, temporal evolutions of cell movement velocity and cytosolic FilGAP concentration are presented. The velocity profile displays a transient increase and decrease with a mean value of approximately 4 μm/min (Figure 2E). Moreover, a time lag of 50–100 s exists in the FilGAP response compared with that of velocity (Figure 2E), presumably due to the bandpass mechanism: FilGAP starts to release when the actin network is largely strained and returns to the FLNa network when the angular strain is mostly recovered. Rac and RhoA operate antagonistically through FilGAP, Rac and RhoA signaling and are switched at the leading edge with a time period comparable to that of FilGAP (Figure 2D). Taken together, the basic model reproduces essential aspects of cell motility in the absence of a stimulus with reasonable distributions of the signaling components.

### 3.2 Model Response to a Gradient Stimulus

**FIGURE 3:**
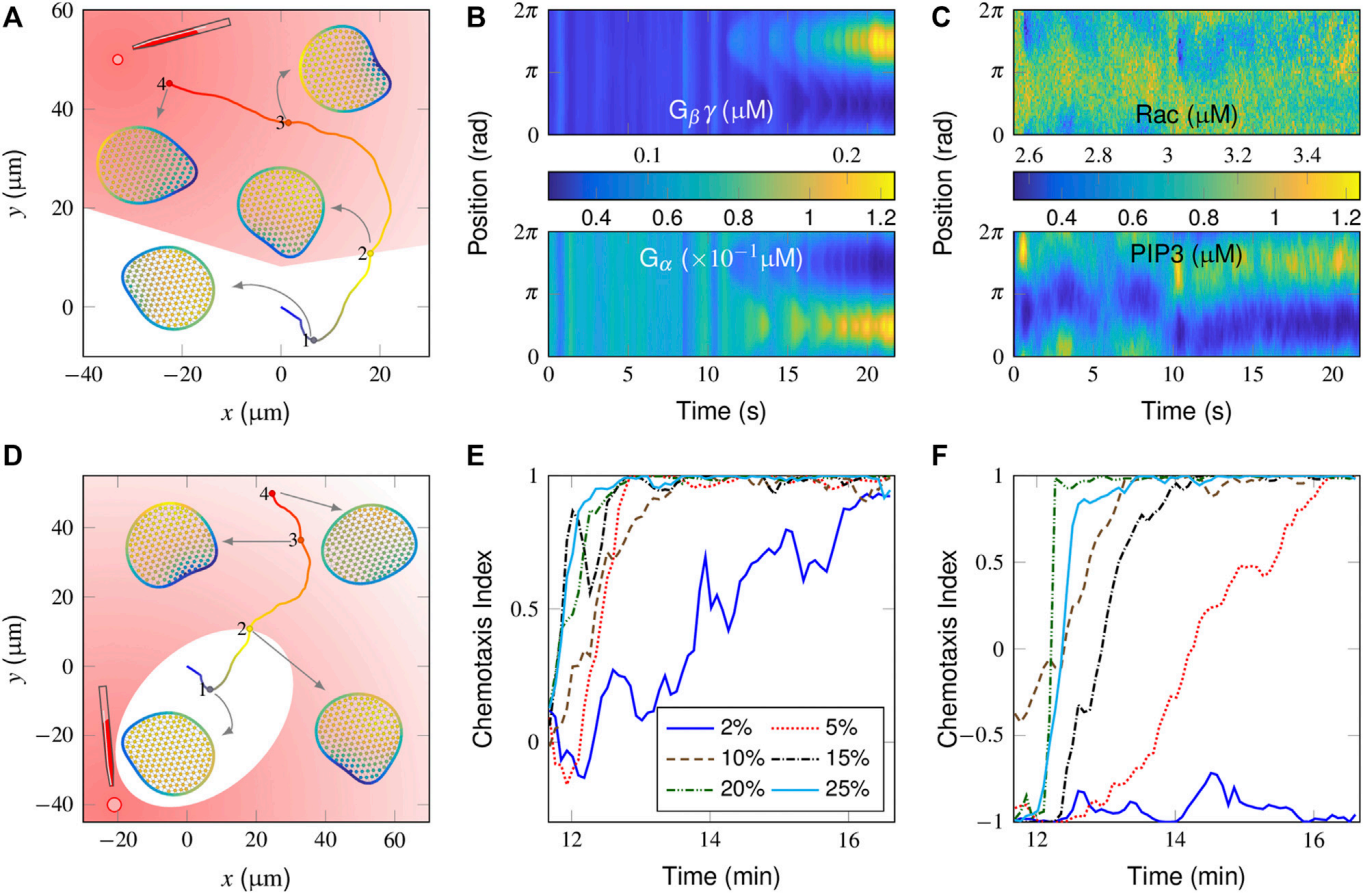
Simulation of chemotaxis behaviors in a “wildtype” cell. **(A)** Trajectory and successive snapshots of cell migration. A gradient stimulus was applied perpendicular to the original migration direction of the cell at *t* = 500 s when the cell reached the “2” position. The cells were not stimulated on the trajectory located in the white area. **(B, C)** Spatiotemporal regulation of the molecules responsible for directional sensing, *i.e.*, G_βγ_ **(B, top)** and G_*α*_ **(B, bottom)**, and for cytoskeletal remodeling, *i.e.*, active Rac **(C, top)** and PIP_3_ **(C, bottom)**. **(D)** Trajectory and successive snapshots of cell migration in response to an opposite gradient stimulus. **(E, F)** Temporal evolution of the chemotaxis index (CI) in response to different gradient stimuli applied either perpendicular **(E)** or opposite **(F)** to the current polarity of the cell. For the definition of CI, please refer to Supplementary Equation S2 in the subsection “Supplementary Material”.

To investigate the chemotaxis behaviors of a “wildtype” (WT) cell, a gradient stimulus with default parameter settings was introduced to a spontaneous migrating cell starting at *t* = 500 s and lasted for an additional 1,600 s. We first tested a case where the initial gradient strength (given in terms of percentage) was only 2% and was applied perpendicular to the polarity the cell had already established (Supplementary Equation S2 describes the percentage as a function of the distance between the cell and the point source). Figure 3A shows the trajectories of cell centroids and a series of successive snapshots of the cell shape colored by PIP_3_ polarity (also see Supplementary Video S2). Clearly, after receiving this gradient stimulus, the cell gradually changed its migration mode from correlated random migration to directional migration by modulating its PIP_3_ polarity.

In Figures 3B,C, the spatiotemporal plots of signaling components in a time period before and after receiving directional guidance are further presented. For the balance-inactivation mechanism (Levine et al., 2006), G_βγ_ and G_*α*_, as activators and inhibitors, respectively, may localize to the membranous regions where the attractant concentration is greatest and weakest, respectively (Figure 3B). The external attractant gradient is thus translated into a spatial bias in Rac activity (Figure 3C, top), which then further regulates PIP_3_. Specifically, the spatiotemporal plot of PIP_3_ (Figure 3C, bottom) clearly shows how a new pseudopod evolved from a pre-existing protrusion. Alternatively, we test the case with the same gradient stimulus but in the opposite direction (*i.e.*, at a 180° relative angle). Here, the plots of the derived cell trajectories and successive snapshots show that the cell is not able to reorient itself to the point source (Figure 3D).

By adjusting the relative position of the point source (Supplementary Figure S1A), we further test how sensitive our model is for differentiating gradient strengths. Figure 3E shows the temporal evolution of the chemotaxis index (CI), derived from gradient stimuli, which are applied perpendicularly to the current polarity. Clearly, the cell responds better to the stronger gradients with higher CI values. At the lowest 2% gradient response, the cell movement tends to be aligned but with considerable variability.

We next considered how a spontaneous migrating cell reacts to the gradients from opposite directions. As already illustrated in Figure 3D, the cell is unable to align itself to a 2% gradient, 180° apart (also see Supplementary Video S3). Correspondingly, the derived CI profile starts from 1 and remains at low values (Figure 3F, blue line). Conversely, the profiles increase from the initial value of -1 to maximum value of +1 at higher gradients, indicating that the cell is aligned preferentially along the direction of the gradient. A further comparison is conducted for the cell movement velocity plot (Supplementary Figure S1B). Note that there is a sudden drop in the velocity after receiving a 5% gradient, applied in an opposite manner, indicating that the cell may return to an almost unpolarized state and be repolarized to the external gradient. In other words, the cell performs a reversal rather than a U-turn to react to an opposite gradient stimulus.

### 3.3 Model Response of Mutant Cells to a Graded Signal

**FIGURE 4:**
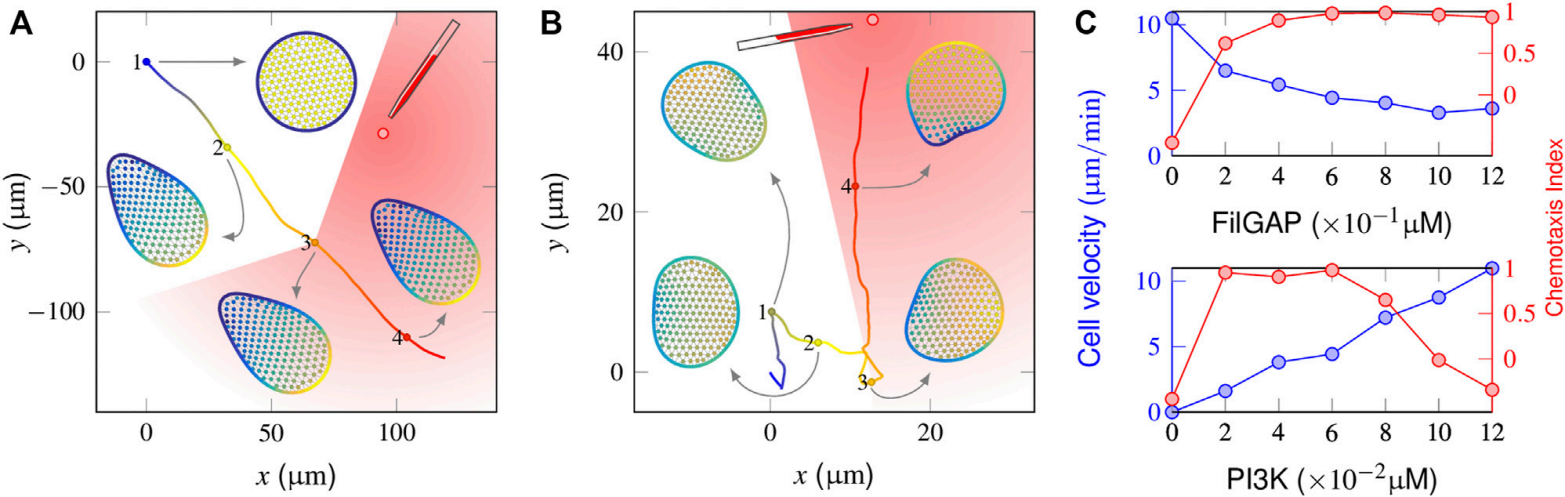
Responses of a mutant cell to a gradient stimulus. **(A)** In the absence of a mechanical inhibition effect (*i.e.*, FilGAP release upon cell deformation), the protrusion front becomes confined, and the cell cannot target itself to the stimulus source (the chemoattractants are released at *t* = 500 s when the cell reaches the “3” position). **(B)** With FilGAP overexpression, the cell responds to the graded stimuli precisely with slow velocity. **(C)** Profiles of average velocity (blue) and the chemotaxis index (red) derived from FilGAP **(top)** and PI3K mutant cells **(bottom)**.

We next considered the chemotaxis behaviors in a number of mutant cells. Applying a 2% graded stimulus perpendicular to a spontaneously migrating FilGAP-abolished cell manifested a complete failure to reorient (Figure 4A and Supplementary Video S4). Spatiotemporal regulation of activator and inhibitor indicated that the cell clearly sensed the stimuli (cf. Figure 4B). However, the amplification of intracellular signaling became too high, causing a freezing state of the initial polarization. In contrast, although a FilGAP-overexpressing cell can respond chemotactically to the same external gradients (Figure 4B and Supplementary Video S5), only a limited number of pseudopods is produced with sluggish motion. The magnitudes of movement velocity and the CI in the late chemotactic stage are presented in the top panel of Figure 4C, wherein the (FilGAP) is varied between these two extremes. Although the cell migrates fast (∼10 μ*m*/min) in the absence of FilGAP, the corresponding CI value is negative, indicating that the cell moves away from the point source. When (FilGAP) increases to 0.12 μM, cellular deformation tends to promote FilGAP release and repress its polarity so that the cell motion is repolarized repeatedly to the external gradient, leading to precise but slow (∼3 μ*m*/min) cell motion. A similar operation is also applied to PI3K mutants by varying (PI3K) from 0 to 0.12 μM while maintaining (FilGAP) at 0.6 μM. As shown in the bottom panel of Figure 4C, the cell is immobilized in the absence of PI3K. In general, the cell movement velocity is increased as (PI3K) increases [the velocity is restricted by the saturation density of F-actin, which is positively correlated with (PIP_3_)]. Having (PI3K) = 0.12 μM leads to an average velocity of 10.3 μm/min but tends to “freeze” single peaks at the same time, reducing the ability of the cell to respond by turning and dropping off the CI value steeply.

Taken together, these numerical simulations indicate that the complementary effect of the two signaling pathways is definitely required for a migrating cell that accurately follows the graded stimuli and reaches its target. Consequently, the most effective strategy for chemotaxis is to establish a fine balance between sufficient feedback for autoamplification that enhances the peaks of PI activity and the mechanical inhibition effect that avoids the overly dramatic kurtosis of those peaks.

### 3.4 Model Responses in the Presence of Obstacles

Under *in vivo* conditions, chemotactic cells must avoid numerous obstacles, *e.g.*, nonchemotactic cells and foreign debris. While the importance of feedback between shape and biochemistry or the spatial perturbation of chemical field at the obstacle’s vicinity has been well discussed (Grima, 2007; Marée et al., 2012), here we focus on how the effects of complementary signaling pathways enable the cell to cope with such challenge.

**FIGURE 5:**
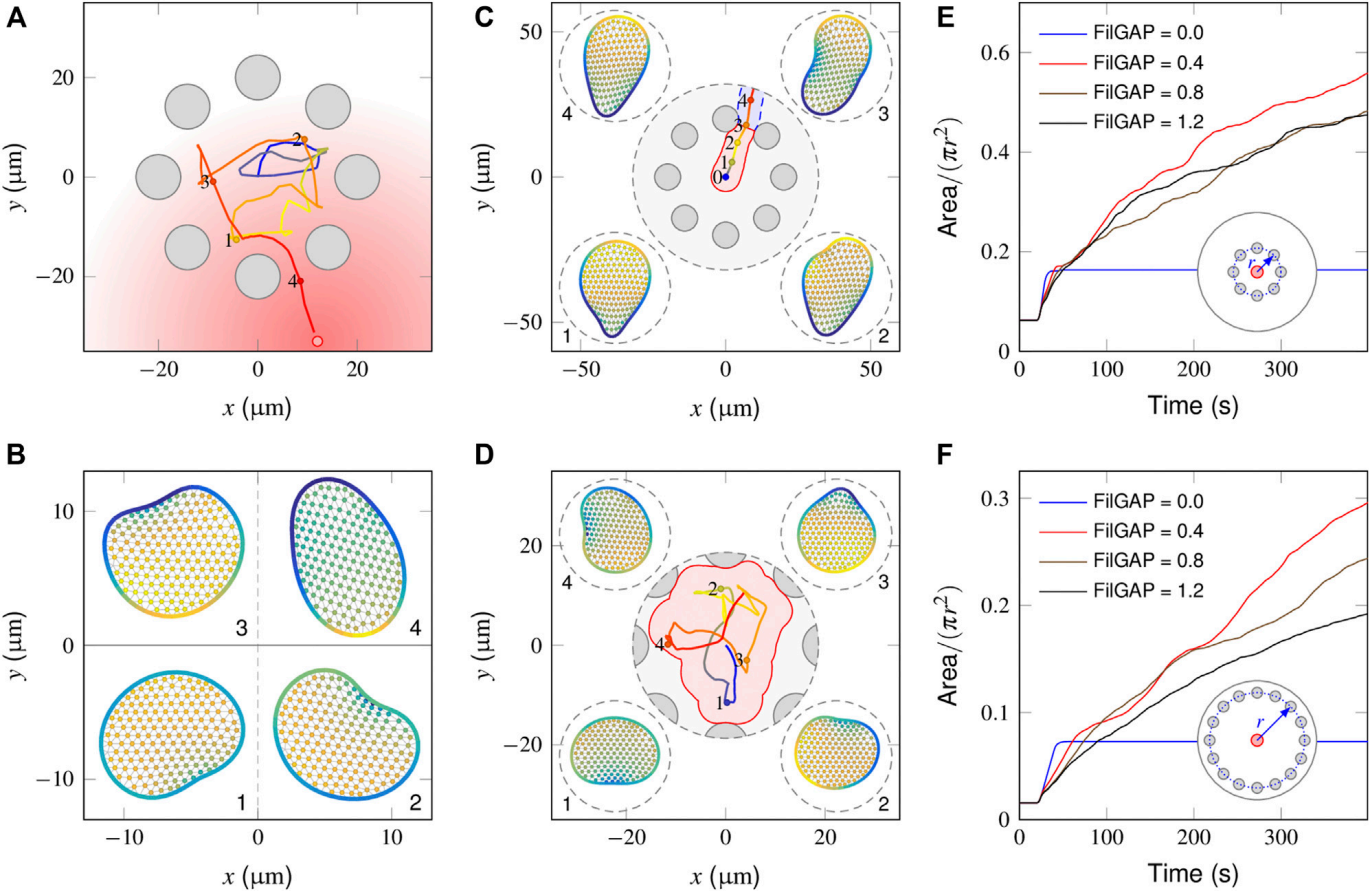
Model responses in the presence of obstacles. **(A)** Cell centroid trajectory during spontaneous (0–500 s) and chemotactic (500–1,500 s) migration within the surrounding steady obstacles (gray circular objects). **(B)** Snapshots of a cell shape at four time points derived from the above cell trajectory plot. **(C)** In the absence of a mechanical inhibition effect, the fully polarized cell glides smoothly along the wall of the obstacle that it encounters and escapes from the region between the two adjacent obstacles, leaving a small search area (pink area). **(D)** In the presence of extremely high inhibition [(FilGAP) ∼1.4 μ*M*], the cell frequently changes its migration direction, achieving a large search area (pink area). **(E, F)** Temporal evolution of the coverage ratio of FilGAP mutant cells within small **(E)** and large **(F)** obstacle surrounding areas. Overexpression of FilGAP can lead to a slow cell moving velocity, resulting in a low searching efficiency, especially regarding a large obstacle surrounding the area.

In Figure 5A, we illustrate how a “wildtype” cell behaves in a local region surrounded by equally spaced obstacles (also see Supplementary Video S6). In the absence of stimuli (0–20 min), since the cell essentially executes a random walk (Figure 2A), the interactions with obstacles abruptly change its migratory direction and produce a shuttle run. At *t* = 20 min, the graded stimulus is applied at the outer side of the obstacle cycle. The trajectory plots indicate that the cell smoothly selects a path and navigates from the interval of two obstacles. Figure 5B shows successive snapshots of cell shape changes during spontaneous (points 1, 2 and 3) and chemotactic migration (point 4). When a spontaneously migrating cell encounters obstacles, its leading edge flattens and extends parallel to the wall. Given that the mechanical strain promotes the extra release of FilGAP, this quickly extinguishes the PIP_3_-rich protrusion fronts and encourages the development of new ones pointing away from the regions of cell-obstacle contact (Figure 5B-2,3). In the presence of chemotactic guidance, however, cell polarity is continually aligned toward the outside of the obstacle region, such that the cell may chemotactically “escape” (Figure 5B-4).

We next considered how FilGAP mutant cells behave in the presence of obstacles and asked how this translates into biological outcomes. In the absence of FilGAP, as explained in Section “Model response of mutant cells to a graded signal”, the cell becomes fully polarized and migrates in a straight trajectory. When such a cell encounters an obstacle, since no extra FilGAP was released to perturb the polarity already established, the cell smoothly moves along the obstacle wall and squeezes out this local region from the interval of two obstacles (Supplementary Video S7), leaving a small coverage area (Figure 5C, pink area). In the presence of an extremely high mechanical inhibition effect, *e.g.*, (FilGAP) = 1.4 μM, a large coverage area can be achieved because the cell displays the correlated random motion endowed by the obstacle confinement (Figure 5D). To quantify the influence of (FilGAP) on the coverage area, we introduced the coverage ratio, *η*, defined as the coverage area divided by the total area of the local region. As seen in Figure 5E, the temporal evolution in the absence of FilGAP shows a monotonic increase with time and reaches a maximum at *t* = 21 s, a time point when the cell escapes from the obstacle area. With an intermediate level of FilGAP, *e.g.*, (FilGAP) = 0.4 μM, the cells move relatively fast, such that the *η* profile manifests an increase with time, leaving a smaller region to be searched. Note that at later stages, the profile slows down because of the overlap effect. With the overexpression of (FilGAP), *e.g.*, (FilGAP) = 1.2 μM, the cell moves slowly at ∼4μm/min , such that the *η* profile manifests a slower increase. We then carried out another set of simulations within a larger obstacle region with a radius of *r* = 40 μm. Clearly, the cell with an intermediate level of FilGAP, *e.g.*, (FilGAP) = 0.4 μM, exhibits the highest searching efficiency, demonstrating that the magnitude of the movement velocity becomes a limiting factor. Taken together, the results in this section imply that the complementary effects of the FLNa-FilGAP and PI-Rho GTPase pathways allow a cell to efficiently search its surrounding area when chasing the stimulus out of the obstacle region.

## 4 Discussion

In this work, we have proposed a mechanochemical coupling model for eukaryotic chemotaxis. Briefly, the model considers that cell motility is controlled by two signaling pathways. In the first or “biochemical” pathway, the spatial effects of Rho GTPase-PIs mediating positive/negative-feedback loops could be invoked either by chemoattractant stimuli or by endogenous stochastic signals. In the second or “biomechanical” pathway, the interplay of FLNa-FilGAP is incorporated in a discrete network of actin filaments. Elucidating their complementary effects in different conditions obtains a generalized view of eukaryotic chemotaxis. First, the cell uses endogenous signals for the stochastic generation of pseudopods without attractant stimuli. The consequent inhibition effect raised from the strain-mediated FilGAP release ensures the occasional modification of an existing protrusion, allowing the cell to explore the local regions. Second, graded attractant stimuli activate the upstream signaling cascades of Rho GTPase that first induce a positional bias of the pseudopods and then have an overwhelming effect on mechanical inhibition, such that, on average, the extending pseudopods are continually oriented toward the gradient. Third, the cell has become trapped within an obstacle-ridden spatial region, manifesting a shuttle run for local explorations and chemotactically “escaping”. To further explain the above insights, three key issues are discussed below: 1) earlier modeling works and core principles for constructing our model, 2) comparisons with experimental data, and 3) the limitations of our model.

### 4.1 Earlier Modeling Works and Core Principles for Constructing Our Model

One theoretical difficulty in simulating chemotaxis is how to adequately abstract and simplify the intracellular signaling cascades. Generally, two different approaches exist. One is the “chemical compass” approach, in which the localized intracellular signals at the point of highest receptor occupancy are analogous to a compass needle pointing toward the source of the chemoattractant. Models belonging to this approach are proposed for protein blocking or exogenous activation experiments. For instance, the LEGI mechanism (Janetopoulos et al., 2004) for gradient sensing is based on the fact that the cytoskeleton is frozen by drugs and the wave-pinning mechanism (Mori et al., 2008) on the measurements of exogenous activation of Rac (Lin et al., 2012). Another is the pseudopod-centered approach, which emphasises the endogenous, autocatalytic growth of pseudopods and allows the unprocessed gradient information to bias multiple points in the cycle (Neilson et al., 2011). While persistent effort is being expended in the field of chemotaxis concerning how to integrate the two approaches (Iglesias and Devreotes, 2008; Van Haastert P. J. M., 2010), one similarity is that they both rely on introducing the activity of an inhibitor. Previously, mechanical factors have been shown to act as inhibitors in many theoretical works, yet how they function in conjunction with migratory cell behaviors has not been fully addressed. A pure reaction-diffusion system in a static cell perimeter was proposed where the feedback between Rac and F-actin was combined with the repression of actin polymerization by membrane tension (Wang et al., 2017). A pure mechanical approach was also presented wherein the protrusive rate of the leading edge depends biphasically on the membrane tension upon a force-balance relationship between protrusion and retraction (He and Ji, 2017). Moreover, a mechano-chemical coupling model was proposed for simulating how a neutrophil enters into a narrow channel when the FLNa-FilGAP pathway is implicitly considered (Wu and Feng, 2015). A similar implicit treatment was also adopted by directly introducing hotspots of Rac activity on the cell membrane (Merchant et al., 2018b).

The core principle for constructing our model is to elaborate the above intricate mechanisms into simplified prototypes that can be readily understood. The bottom of Figure 1B shows the bidirectional molecular transport mechanism, which allows a cell to use endogenous signals for the highly ordered extension of pseudopods. Its upstream Rho GTPase regulation and initial signaling processing modules together act as a compass for amplifying extracellular guidance messenger and translate to the bottom module, such that an all-or-none distribution pattern of intracellular signaling components can be achieved. At this stage, the modeled cell can be regarded as a multilayered dynamic seesaw, which only contains biochemical pathways. One recurrent question is that although a cell may easily produce initial polarity and thus directional movement, either by stochastic noise or by guided attractant stimuli, it can only reply to a secondary stimulus with a steeper gradient (Feng et al., 2018). We therefore presume that, in physics, the role of the FLNa-FilGAP pathway is to serve as a brake, as depicted by two springs, into the dynamic seesaw system (Figure 1B, insert). As a result, the seesaw might fluctuate with perturbation, corresponding to the correlated random migration of a cell. However, the dynamic seesaw in the presence of external guidance gradually biases toward the direction of guidance, despite the brake still working, which is essentially analogous to altering the cell movement direction in response to directional cues.

### 4.2 Comparison With Experimental Data

Our simulations might also provide rational explanations at the molecular level for a set of seemingly controversial measurements at the cellular level. The first set of simulations (Figure 1 and Figure 2) were motivated by the data published by Van Haastert PJ. (2010) and Bosgraaf and Van Haastert (2009), where pseudopodia formation by *Dictyostelium* cells was analyzed, revealing that pseudopods are frequently formed in two ways. First, pseudopod splitting occurs very frequently, alternating to the right and left at a relatively small angle of ∼55° and leading to a persistent zig-zag trajectory. Second, the cells may also extend pseudopodia from the areas in which the cells are not previously active, described as *de novo* pseudopodia, inducing a random turn of the cells. In starved *Dictyostelium* cells, the probability of extending a *de novo* pseudopod is 10-fold lower than that of pseudopod splitting (Van Haastert PJ., 2010). Upon our simulations in mutant cells, such behaviors could be accounted for as an adaptation strategy for evaluating the efficiency in searching the local environment and could be achieved by adjusting the strength of two signaling pathways. The second set of simulations (Figure 3 and Figure 5) was intended to compare the data published by Andrew and Insall (2007). In their work, the quantitative analysis of pseudopod generation was carried out for chemotaxis in shallow gradients in multiple cell types, including *Dictyostelium* cells and neutrophils, indicating that 90% of new protrusions were derived from pre-existing ones and rarely emerged from previously pseudopod-free regions of the cell. Upon our simulations, the great difference in the probability of choosing two modes is caused by both the direction and the steepness of the gradient stimulus. Because a cell manifests a proper balance between two signaling pathways, it shows high sensitivity to a shallow gradient stimulus applied from its direction of advancement because such stimuli may continue modifying the existing pseudopod of the cell. However, if the stimulus is applied in an opposite manner, the modification rate may be too slow to guide. In this regard, only a steeper gradient is effective, as its effect is strong enough to produce a new pseudopod directly.

### 4.3 Limitations of Our Model

First, to avoid introducing too many details at once, our model did not include the signaling cascades for establishing cell-substrate adhesion. While the spatiotemporal regulations of the signaling cascades responsible for cytoskeleton remodeling are widely conserved (Parent, 2004; Van Haastert and Devreotes, 2004), those for cellular adhesion vary significantly from one cell type to another. For instance, rapidly moving cells such as neutrophils (speeds up to 10–2 μm/min) only establish small and transient focal complexes (Holmes and Edelsteinkeshet, 2012). In contrast, slowly moving fibroblasts (speeds of only 1 μm/min) are characterized by strong focal adhesion (Holmes and Edelsteinkeshet, 2012). The establishment of cell-substrate adhesion itself is also a mechano-sensing process (Bershadsky et al., 2006; Giannone and Sheetz, 2006). Further incorporating the models with the assembly and disassembly of cell-substrate adhesion enables our model to provide a possible explanation regarding why there are unique features between different cells.

Second, the current model tends to focus on explaining transient signaling activity on the membrane, for the production and competition of pseudopods, and regarding the role of pseudopod dynamics in chemotaxis. Accordingly, the details of the cytoskeletal remodeling process are omitted, and the signaling activity is implicitly linked to forces causing protrusion and retraction. Cytoskeletal remodeling is likely governed by the polarities of signaling molecule distributions (refer to Supplementary Material), but their intercorrelation is far more sophisticated. A variety of mechanisms, including G-actin treadmilling, dendritic nucleation at the leading edge, and vesicle delivery by microtubules, may function together to regulate the dynamic patterns of actin-myosin flow (Rubinstein et al., 2009; Wolgemuth et al., 2010).

Finally, our model is a simple but efficient numerical tool for mechano-chemical coupling problems. This method could be applicable in the field of collective cell migration (Feng et al., 2018), wherein the position-dependent cell directionality and speed are of greatest concern. Further improvements could be achieved by incorporating the particle-spring model with the immersed boundary (IB) method (Dallon et al., 2009; Wu and Feng, 2015), such that the integrated method could be used to decipher biological processes involving fluid-solid interactions. Nevertheless, the efficient treatment of signaling cascades in our model also requires an accurate account of the cell morphology. In this regard, the CPM method is a more suitable platform (Marée et al., 2012).

## Data Availability

The original contributions presented in the study are included in the article/Supplementary Material, further inquiries can be directed to the corresponding authors.

## References

[B1] Andasari V. Lü D. Swat M. Feng S. Spill F. Chen L. (2018). Computational Model of Wound Healing: Egf Secreted by Fibroblasts Promotes Delayed Re-Epithelialization of Epithelial Keratinocytes. Integr. Biol. 10, 605–634. 10.1039/c8ib00048d PMC6571173 30206629

[B2] Andrew N. Insall R. H. (2007). Chemotaxis in Shallow Gradients Is Mediated Independently of Ptdins 3-Kinase by Biased Choices Between Random Protrusions. Nat. Cell Biol. 9, 193–200. 10.1038/ncb1536 17220879

[B3] Bershadsky A. Kozlov M. Geiger B. (2006). Adhesion-Mediated Mechanosensitivity: a Time to Experiment, and a Time to Theorize. Curr. Opin. Cell Biol. 18, 472–481. 10.1016/j.ceb.2006.08.012 16930976

[B4] Billadeau D. D. (2008). Pten Gives Neutrophils Direction. Nat. Immunol. 9, 716–718. 10.1038/ni0708-716 18563079

[B5] Bosgraaf L. Van Haastert P. J. (2009). The Ordered Extension of Pseudopodia by Amoeboid Cells in the Absence of External Cues. Plos One 4, e5253. 10.1371/journal.pone.0005253 19384419 PMC2668753

[B6] Castellani V. (2013). Building Spinal and Brain Commissures: Axon Guidance at the Midline. ISRN Cell Biol. 2013, 1–21. 10.1155/2013/315387

[B7] Comer F. I. Parent C. A. (2002). PI 3-Kinases and PTEN. Cell 109, 541–544. 10.1016/s0092-8674(02)00765-1 12062096

[B8] Dallon J. C. Newren E. Hansen M. D. (2009). Using a Mathematical Model of Cadherin-Based Adhesion to Understand the Function of the Actin Cytoskeleton. Phys. Rev. E Stat. Nonlin. Soft Matter Phys. 79, 031918. 10.1103/PhysRevE.79.031918 19391982

[B9] Danuser G. Allard J. Mogilner A. (2013). Mathematical Modeling of Eukaryotic Cell Migration: Insights Beyond Experiments. Annu. Rev. Cell Dev. Biol. 29, 501–528. 10.1146/annurev-cellbio-101512-122308 23909278 PMC4148455

[B10] Devreotes P. Janetopoulos C. (2003). Eukaryotic Chemotaxis: Distinctions Between Directional Sensing and Polarization. J. Biol. Chem. 278, 20445–20448. 10.1074/jbc.r300010200 12672811

[B11] Ehrlicher A. J. Nakamura F. Hartwig J. H. Weitz D. A. Stossel T. P. (2011). Mechanical Strain in Actin Networks Regulates Filgap and Integrin Binding to Filamin a. Nature 478, 260–263. 10.1038/nature10430 21926999 PMC3204864

[B12] Feng S. Zhou L. Zhang Y. Lü S. Long M. (2018). Mechanochemical Modeling of Neutrophil Migration Based on Four Signaling Layers, Integrin Dynamics, and Substrate Stiffness. Biomech. Model. Mechanobiol. 17, 1611–1630. 10.1007/s10237-018-1047-2 29968162

[B13] Franca-Koh J. Devreotes P. N. (2004). Moving Forward: Mechanisms of Chemoattractant Gradient Sensing. Physiology 19, 300–308. 10.1152/physiol.00017.2004 15381759

[B14] Fukata M. Nakagawa M. Kaibuchi K. (2003). Roles of Rho-Family Gtpases in Cell Polarisation and Directional Migration. Curr. Opin. Cell Biol. 15, 590–597. 10.1016/s0955-0674(03)00097-8 14519394

[B15] Gallage H. N. P. Saha S. C. Gu Y. (2013). Deformation of a Single Red Blood Cell in a Microvessel. ANZIAM J. 55, C64–C79. 10.21914/anziamj.v55i0.7828

[B16] Gambardella L. Vermeren S. (2013). Molecular Players in Neutrophil Chemotaxis-Focus on PI3K and Small GTPases. J. Leukoc. Biol. 94, 603–612. 10.1189/jlb.1112564 23667166

[B17] Giannone G. Sheetz M. P. (2006). Substrate Rigidity and Force Define Form through Tyrosine Phosphatase and Kinase Pathways. Trends Cell Biol. 16, 213–223. 10.1016/j.tcb.2006.02.005 16529933

[B18] Grima R. (2007). Directed Cell Migration in the Presence of Obstacles. Theor. Biol. Med. Model. 4, 2. 10.1186/1742-4682-4-2 17227579 PMC1797164

[B19] He S. Ji B. (2017). Mechanics of Cell Mechanosensing in Protrusion and Retraction of Lamellipodium. ACS Biomater. Sci. Eng. 3, 2943–2953. 10.1021/acsbiomaterials.6b00539 33418714

[B20] Holmes W. R. Edelstein-Keshet L. (2012). A Comparison of Computational Models for Eukaryotic Cell Shape and Motility. Plos Comput. Biol. 8, e1002793. 10.1371/journal.pcbi.1002793 23300403 PMC3531321

[B21] Iglesias P. A. Devreotes P. N. (2008). Navigating Through Models of Chemotaxis. Curr. Opin. Cell Biol. 20, 35–40. 10.1016/j.ceb.2007.11.011 18207721

[B22] Janetopoulos C. Ma L. Devreotes P. N. Iglesias P. A. (2004). Chemoattractant-Induced Phosphatidylinositol 3,4,5-trisphosphate Accumulation Is Spatially Amplified and Adapts, Independent of the Actin Cytoskeleton. Pnas. 101, 8951–8956. 10.1073/pnas.0402152101 15184679 PMC428453

[B23] Kang J. Puskar K. M. Ehrlicher A. J. Leduc P. R. Schwartz R. S. (2015). Structurally Governed Cell Mechanotransduction Through Multiscale Modeling. Sci. Rep. 5, 8622. 10.1038/srep08622 25722249 PMC4342557

[B24] Kolaczkowska E. Kubes P. (2013). Neutrophil Recruitment and Function in Health and Inflammation. Nat. Rev. Immunol. 13, 159–175. 10.1038/nri3399 23435331

[B25] Levine H. Kessler D. A. Rappel W.-J. (2006). Directional Sensing in Eukaryotic Chemotaxis: A Balanced Inactivation Model. Proc. Natl. Acad. Sci. 103, 9761–9766. 10.1073/pnas.0601302103 16782813 PMC1502527

[B26] Lin B. Holmes W. R. Wang C. J. Ueno T. Harwell A. Edelstein-Keshet L. (2012). Synthetic Spatially Graded Rac Activation Drives Cell Polarization and Movement. Proc. Natl. Acad. Sci. U S A. 109, E3668–E3677. 10.1073/pnas.1210295109 23185021 PMC3535611

[B27] Lipshtat A. Jayaraman G. He J. C. Iyengar R. (2010). Design of Versatile Biochemical Switches that Respond to Amplitude, Duration, and Spatial Cues. Proc. Natl. Acad. Sci. 107, 1247–1252. 10.1073/pnas.0908647107 20080566 PMC2824311

[B28] Marée A. F. M. Grieneisen V. A. Edelstein-Keshet L. (2012). How Cells Integrate Complex Stimuli: the Effect of Feedback From Phosphoinositides and Cell Shape on Cell Polarization and Motility. Plos Comput. Biol. 8, e1002402. 10.1371/journal.pcbi.1002402 22396633 PMC3291540

[B29] Merchant B. Edelstein-Keshet L. Feng J. J. (2018a). A Rho-Gtpase Based Model Explains Spontaneous Collective Migration of Neural Crest Cell Clusters. Developmental Biol. 444, S262–S273. 10.1016/j.ydbio.2018.01.013 29366821

[B30] Merchant B. Edelstein-Keshet L. Feng J. J. (2018b). A Rho-Gtpase Based Model Explains Spontaneous Collective Migration of Neural Crest Cell Clusters. Dev. Biol. 444 Suppl 1, S262. 10.1016/j.ydbio.2018.01.013 29366821

[B31] Mori Y. Jilkine A. Edelstein-Keshet L. (2008). Wave-Pinning and Cell Polarity From a Bistable Reaction-Diffusion System. Biophysical J. 94, 3684–3697. 10.1529/biophysj.107.120824 PMC2292363 18212014

[B32] Nakamura F. (2013). Filgap and its Close Relatives: a Mediator of Rho-Rac Antagonism That Regulates Cell Morphology and Migration. Biochem. J. 453, 17–25. 10.1042/bj20130290 23763313

[B33] Neilson M. P. Veltman D. M. van Haastert P. J. M. Webb S. D. Mackenzie J. A. Insall R. H. (2011). Chemotaxis: A Feedback-Based Computational Model Robustly Predicts Multiple Aspects of Real Cell Behaviour. Plos Biol. 9, e1000618. 10.1371/journal.pbio.1000618 21610858 PMC3096608

[B34] Onsum M. Rao C. V. (2005). A Mathematical Model for Neutrophil Gradient Sensing and Polarization. Plos Comput. Biol. 3, e36–450. 10.1371/journal.pcbi.0030036 PMC1828701 17367201

[B35] Parent C. A. (2004). Making All the Right Moves: Chemotaxis in Neutrophils and dictyostelium. Curr. Opin. Cell Biol. 16, 4–13. 10.1016/j.ceb.2003.11.008 15037299

[B36] Paul R. Heil P. Spatz J. P. Schwarz U. S. (2008). Propagation of Mechanical Stress Through the Actin Cytoskeleton Toward Focal Adhesions: Model and experiment. Biophysical J. 94, 1470–1482. 10.1529/biophysj.107.108688 PMC2212708 17933882

[B37] Petrie R. J. Doyle A. D. Yamada K. M. (2009). Random Versus Directionally Persistent Cell Migration. Nat. Rev. Mol. Cell Biol. 10, 538–549. 10.1038/nrm2729 19603038 PMC2752299

[B38] Plotnikov S. V. Pasapera A. M. Sabass B. Waterman C. M. (2012). Force Fluctuations Within Focal Adhesions Mediate Ecm-Rigidity Sensing to Guide Directed Cell Migration. Cell 151, 1513–1527. 10.1016/j.cell.2012.11.034 23260139 PMC3821979

[B39] Polacheck W. J. Zervantonakis I. K. Kamm R. D. (2013). Tumor Cell Migration in Complex Microenvironments. Cell Mol. Life Sci. 70, 1335–1356. 10.1007/s00018-012-1115-1 22926411 PMC3557537

[B40] Raftopoulou M. Hall A. (2004). Cell Migration: Rho Gtpases Lead the Way. Developmental Biol. 265, 23–32. 10.1016/j.ydbio.2003.06.003 14697350

[B41] Rottner K. Stradal T. E. (2011). Actin Dynamics and Turnover in Cell Motility. Curr. Opin. Cell Biol. 23, 569–578. 10.1016/j.ceb.2011.07.003 21807492

[B42] Rubinstein B. Fournier M. F. Jacobson K. Verkhovsky A. B. Mogilner A. (2009). Actin-Myosin Viscoelastic Flow in the Keratocyte Lamellipod. Biophysical J. 97, 1853–1863. 10.1016/j.bpj.2009.07.020 PMC2756368 19804715

[B43] Shi C. Huang C. H. Devreotes P. N. Iglesias P. A. (2013). Interaction of Motility, Directional Sensing, and Polarity Modules Recreates the Behaviors of Chemotaxing Cells. Plos Comput. Biol. 9, e1003122. 10.1371/journal.pcbi.1003122 23861660 PMC3701696

[B44] Stephens L. Milne L. Hawkins P. (2008). Moving Towards a Better Understanding of Chemotaxis. Curr. Biol. 18, R485–R494. 10.1016/j.cub.2008.04.048 18522824

[B45] Van Haastert P. J. (2010b). A Model for a Correlated Random Walk Based on the Ordered Extension of Pseudopodia. Plos Comput. Biol. 6, e1000874. 10.1371/journal.pcbi.1000874 20711349 PMC2920832

[B46] Van Haastert P. J. M. (2010a). Chemotaxis: Insights From the Extending Pseudopod. J. Cell Sci. 123, 3031–3037. 10.1242/jcs.071118 20810783

[B47] Van Haastert P. J. M. Devreotes P. N. (2004). Chemotaxis: Signalling the Way Forward. Nat. Rev. Mol. Cell Biol. 5, 626–634. 10.1038/nrm1435 15366706

[B48] Wang F. Herzmark P. Weiner O. D. Srinivasan S. Servant G. Bourne H. R. (2002). Lipid Products of Pi(3)ks Maintain Persistent Cell Polarity and Directed Motility in Neutrophils. Nat. Cell Biol. 4, 513–518. 10.1038/ncb810 12080345

[B49] Wang W. Tao K. Wang J. Yang G. Ouyang Q. Wang Y. (2017). Exploring the Inhibitory Effect of Membrane Tension on Cell Polarization. Plos Comput. Biol. 13, e1005354. 10.1371/journal.pcbi.1005354 28135277 PMC5305267

[B50] Weiner O. D. Neilsen P. O. Prestwich G. D. Kirschner M. W. Cantley L. C. Bourne H. R. (2002). A PtdInsP3- and Rho GTPase-Mediated Positive Feedback Loop Regulates Neutrophil Polarity. Nat. Cell Biol. 4, 509–513. 10.1038/ncb811 12080346 PMC2823287

[B51] Welf E. S. Haugh J. M. (2011). Signaling Pathways That Control Cell Migration: Models and Analysis. Wires Syst. Biol. Med. 3, 231–240. 10.1002/wsbm.110 PMC3052860 21305705

[B52] Wolgemuth C. W. Stajic J. Mogilner A. (2010). Redundant Mechanisms for Stable Cell Locomotion Revealed by Minimal Models. Biophys. J. 101, 545–553. 10.1016/j.bpj.2011.06.032 PMC3145291 21806922

[B53] Wong K. Pertz O. Hahn K. Bourne H. (2006). Neutrophil Polarization: Spatiotemporal Dynamics of Rhoa Activity Support a Self-Organizing Mechanism. Proc. Natl. Acad. Sci. 103, 3639–3644. 10.1073/pnas.0600092103 16537448 PMC1450135

[B54] Wu T. Feng J. J. (2015). Modeling the Mechanosensitivity of Neutrophils Passing Through a Narrow Channel. Biophysical J. 109, 2235–2245. 10.1016/j.bpj.2015.10.032 PMC4675883 26636935

[B55] Yang L. Effler J. C. Kutscher B. L. Sullivan S. E. Robinson D. N. Iglesias P. A. (2008). Modeling Cellular Deformations Using the Level Set Formalism. BMC Syst. Biol. 2, 68. 10.1186/1752-0509-2-68 18652669 PMC2535594

