# Supplementary figures and images for: Two Complementary Signaling Pathways Depict Eukaryotic Chemotaxis: A Mechanochemical Coupling Model
Source: Front Cell Dev Biol. 2021 Nov 17;9:786254. doi: 10.3389/fcell.2021.786254 (PMC8635958; doi:10.3389/fcell.2021.786254)

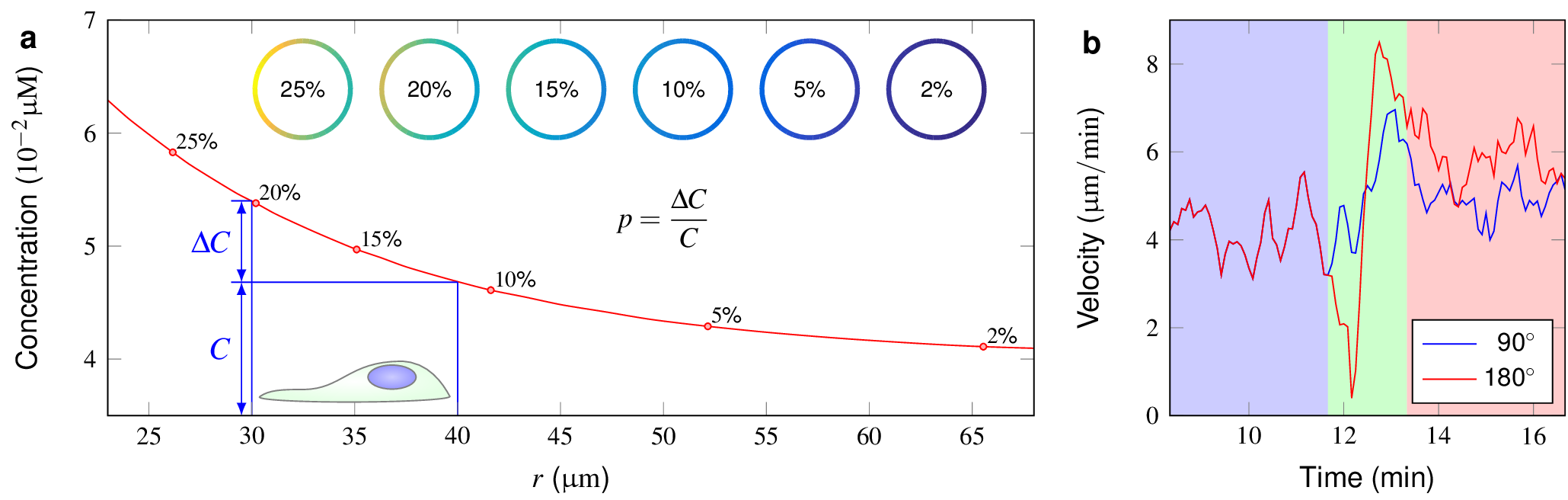

Supplement: Supplementary file 2 [file Image1.TIFF]

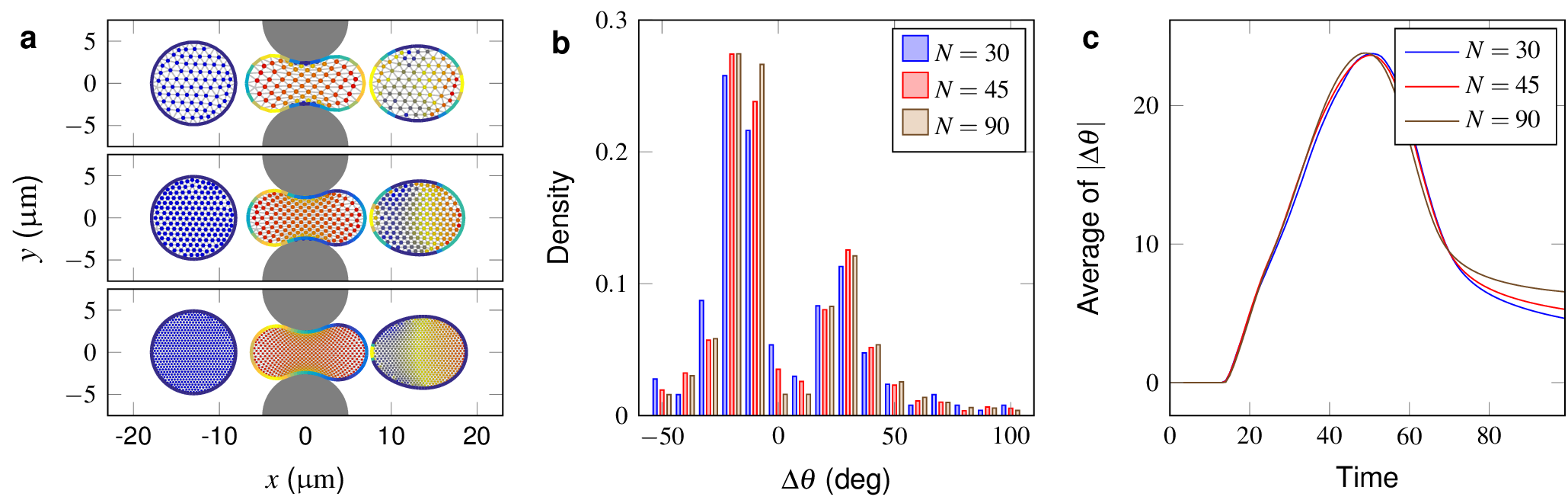

Supplement: Supplementary file 7 [file Image2.TIFF]
