# Supplementary material for: Two Complementary Signaling Pathways Depict Eukaryotic Chemotaxis: A Mechanochemical Coupling Model
Source: Front Cell Dev Biol. 2021 Nov 17;9:786254. doi: 10.3389/fcell.2021.786254 (PMC8635958; doi:10.3389/fcell.2021.786254)
Supplement: Supplementary file 4 [file DataSheet1.pdf]

frontiers

Supplementary Material

1 SUPPLEMENTAL FIGURES

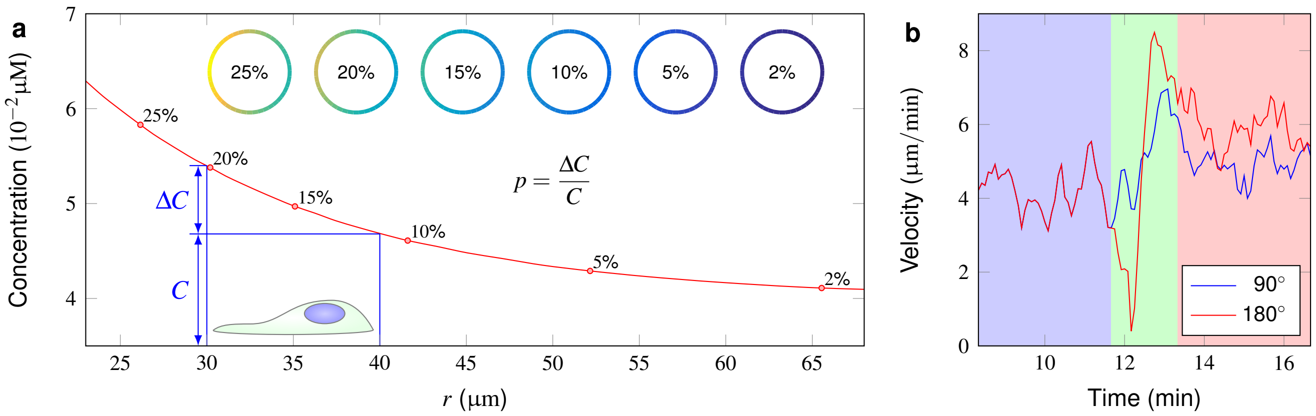

Figure S1. Responses of the modeled cell to a gradient stimulus. **(A)** Gradient profile as a function of the distance (r) between the cell and the stimulus source. Varying r from approximately 65 to 25 µm results in a 2% to 25% change (p). Here, C and ΔC denote the lowest concentration and increment, respectively. **(B)** Velocity profile in response to an initial 5% change in the stimulus gradient. Accordingly, the cell takes spontaneous (purple), adaptable (green), and directional motion (pink).

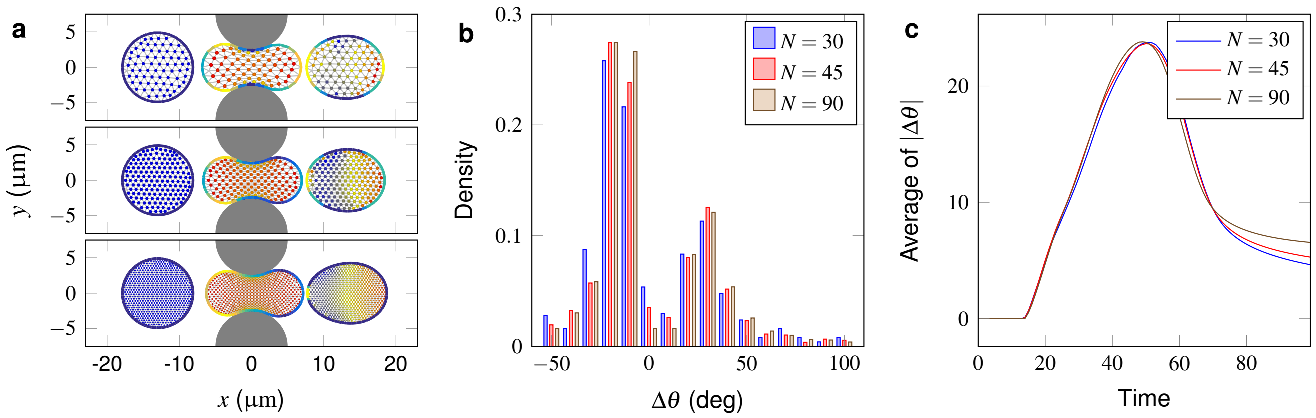

Figure S2. Robustness analysis of numerical simulations. **(A)** Snapshots of the shape changes of the modeled cells with $N = 30$ (upper), 45 (middle) and 90 (lower) membrane nodes passing though the interval of two obstacles. **(B)** Distributions of angular change of the lamellipodial meshwork at a single time point when the cells arrive at the center of the obstacle interval. **(C)** Temporal evolution of the average angular change ($\Delta\theta$) at different mesh densities of 30, 45, and 90.

2 SUPPLEMENTAL VIDEOS

- **Video S1** Correlated random migration of a "wildtype" cell.
- **Video S2** Chemotaxis behaviors in a "wildtype" cell in response to a lateral gradient stimulus.
- **Video S3** Chemotaxis behaviors in a "wildtype" cell in response to an opposite gradient stimulus.

- **Video S4** Responses of a cell without FilGAP to a gradient stimulus.
- **Video S5** Responses of a FilGAP overexpression cell to a gradient stimulus.
- **Video S6** A "wildtype" cell behaves in a local region surrounded by equally spaced obstacles before and after chemoattractant stimuli.
- **Video S7** A cell without FilGAP behaves in a local region surrounded by equally spaced obstacles.

3 MODEL EQUATIONS

3.1 Derivation of the chemoattractant field

The production, diffusion, and degradation of a chemoattractant molecule can be described by

$$\frac{\partial C}{\partial t} = D_s \nabla^2 C - k_d C + k_p, \tag{S1}$$

where $C(r, t)$ is the concentration of the molecule at distance r from the point source at time t, D_s is the diffusion coefficient of the chemoattractants, k_d is the degradation rate, and k_p is the production rate. The solution of this equation has been given by [22]:

$$\begin{aligned} C(r,t) =& \frac{k_p}{k_d}\frac{L}{\lambda}\frac{\cosh\big((L-r)/\lambda\big)}{\sinh(L/\lambda)} \\ & - k_p \sum_{n=-\infty}^{\infty} \tau_n \cos\Big(n\pi\frac{\tau}{L}\Big)\, \mathrm{e}^{-t/\tau_n}, \end{aligned} \tag{S2}$$

where λ is the space constant, $\lambda = \sqrt{D_s/k_d}$, and τ_n is the time constant where $\tau_n = 1/(k_d + n^2\pi D/L)$.

Given a baseline concentration (C_b), the percentage change in the chemoattractant concentration (f) across a small distance $2R$ (R is the radius of the cell) can be defined as:

$$f = \frac{C(x_0 - R) - C(x_0 + R)}{C(x_0 + R) + C_b} \tag{S3}$$

To achieve a desired f value, the distance between the initial position of the cell and the point source (x_0) can be calculated straightforwardly,

$$\begin{aligned} x_0 =& \lambda \ln \Big(\mathrm{e}^{L/\lambda} \sqrt{(f+1)(1+\beta^2)\gamma^2 + C_b f^2 \beta} \\ & \overline{-(f+1)^2\gamma^2\beta - C_b f \mathrm{e}^{(R+L)/\lambda}} \Big) \\ & - \lambda \ln \big(\gamma\big((1+f)\beta - 1\big)\big) \end{aligned} \tag{S4}$$

where $\gamma = C_b L/(k_d \lambda) \cdot 1/\sinh(L/\lambda)$ and $\beta = \mathrm{e}^{2R/\lambda}$.

3.2 Initial signaling processing

Local GPCR occupancy mirrors the local chemoattractant concentration. Hereafter, a balanced-inactivation mechanism [11] is introduced to mimic the initial signaling process. This mechanism involves three interacting steps: (i) the local receptor occupancy level ([RL]) drives the production of membrane-bound species A and cytosolic species I at equal rates, k_s; (ii) the cytosolic species diffuses inside the cell

and attaches itself to the membrane at a rate k_I and becomes the membrane-anchored species I_m; and (iii) both species A and I_m inactivate each other with a rate k_i, and A and I_m spontaneously degrade at rates δ_A and δ_I, respectively. The system equations can be written as

$$\frac{\partial A}{\partial t} = D_{\mathrm{m}} \nabla^2 A + k_{\mathrm{s}}[\mathrm{RL}] - \delta_{\mathrm{A}} A - k_{\mathrm{i}} A I_{\mathrm{m}}, \tag{S5}$$

$$\frac{\partial I_{\mathrm{m}}}{\partial t} = D_{\mathrm{m}} \nabla^2 I_{\mathrm{m}} + k_{\mathrm{I}} I - \delta_{\mathrm{I}} I_{\mathrm{m}} - k_{\mathrm{i}} A I_{\mathrm{m}}, \tag{S6}$$

$$\frac{\partial I}{\partial t} = D_{\mathrm{c}} \nabla^2 I, \tag{S7}$$

with the boundary condition

$$D_{\mathrm{c}} \frac{\partial I}{\partial n} = k_{\mathrm{s}}[\mathrm{RL}] - k_{\mathrm{I}} I. \tag{S8}$$

3.3 Bidirectional molecular transport

Spatiotemporal regulation of $\mathrm{PIP_3}$ (P_3) and $\mathrm{PIP_2}$ (P_2) forms the core of the bidirectional molecular transport mechanism, which is described by the following equations:

$$\frac{\partial P_3}{\partial t} = D_{\mathrm{m}} \nabla^2 P_3 + k_{\mathrm{cat}}^{\mathrm{PI3K}} \left(\frac{H_{\mathrm{m}} P_2 \tilde{R}}{k_{\mathrm{M}}^{\mathrm{PI3K}} + P_2} \right) - k_{\mathrm{cat}}^{\mathrm{PTEN}} \left(\frac{T_{\mathrm{m}} P_3 \tilde{\rho}}{k_{\mathrm{M}}^{\mathrm{PTEN}} + P_3} \right), \tag{S9}$$

$$\frac{\partial P_2}{\partial t} = D_{\mathrm{m}} \nabla^2 P_2 - k_{\mathrm{cat}}^{\mathrm{PI3K}} \left(\frac{H_{\mathrm{m}} P_2 \tilde{R}}{k_{\mathrm{M}}^{\mathrm{PI3K}} + P_2} \right) + k_{\mathrm{cat}}^{\mathrm{PTEN}} \left(\frac{T_{\mathrm{m}} P_3 \tilde{\rho}}{k_{\mathrm{M}}^{\mathrm{PTEN}} + P_3} \right), \tag{S10}$$

where $\tilde{R} = \min\{R/R_{\max},\, 1\}$ and $\tilde{\rho} = \min\{\rho/\rho_{\max},\, 1\}$. In Eq. S9, the first term on the right-hand side accounts for $\mathrm{PIP_3}$ diffusion, the second accounts for $\mathrm{PIP_3}$ production due to membrane-bound PI3K (Hm) acting on $\mathrm{PIP_2}$, and the third accounts for $\mathrm{PIP_3}$ diminution due to membrane-bound PTEN acting on $\mathrm{PIP_3}$. The parameters $k_{\mathrm{cat}}^{\mathrm{PI3K}}$ ($k_{\mathrm{cat}}^{\mathrm{PTEN}}$) and $k_{\mathrm{M}}^{\mathrm{PI3k}}$ ($k_{\mathrm{M}}^{\mathrm{PTEN}}$) are based on the steady state levels of PIs. $\tilde{R}$ ($\tilde{\rho}$) is the normalized factor reflecting the effect of Rac (Rho) activity on PI3K (PTEN) activation, and $R_{\max}$ ($\rho_{\max}$) acts as a constant for Rac (RhoA) activity. If $\tilde{R}$ ($\tilde{\rho}$) is greater than $R_{\max}$ ($\rho_{\max}$), the activity of Rac (RhoA) is no longer a limiting factor for PI3K (PTEN) activation, and $\tilde{R}$ ($\tilde{\rho}$) then equals unity. Eq. S10 describes the $\mathrm{PIP_2}$ dynamics. Similarly, the first term on the right accounts for $\mathrm{PIP_2}$ diffusion, the second accounts for $\mathrm{PIP_2}$ production from $\mathrm{PIP_3}$ *via* membrane-bound PTEN (T_{m}), and the third accounts for the reduction of $\mathrm{PIP_2}$ into $\mathrm{PIP_3}$ *via* membrane-bound PI3K.

3.4 Membrane mechanics

The elastic energy stored in the springs due to stretching or compression is given by

$$E_{\mathrm{l}} = \frac{1}{2} \sum_{i=1}^{n} K_{\mathrm{l}} \left(\frac{l_i - l_0}{l} \right)^2, \tag{S11}$$

where l_i is the length of the ith spring that is iterated at every step, l_0 is the relaxed (zero-force) length, and K_l is the effective stiffness constant of the spring. The elastic energy stored in the spring due to bending is given by

$$E_\mathrm{b} = \frac{1}{2}\sum_{i=1}^{n} K_\mathrm{b}\tan^2\left(\frac{\theta_i - \theta_0}{2}\right), \tag{S12}$$

where θ_i is the angle of the ith spring that is iterated at every step, θ_0 is the relaxed (zero-force) angle, and K_b is the spring constant for bending. In addition, an area constraint is also applied to ensure that the cell area is conserved within 1% during the simulation. This is implemented *via* an energetic penalty as follows:

$$E_\mathrm{s} = \frac{1}{2}K_\mathrm{s}\left(\frac{s - s_0}{s}\right)^2 \tag{S13}$$

where s and s_0 are the instantaneous and equivalent areas of the cell, respectively, and K_s is the penalty coefficient.

The total elastic energy (E_m) of the cell membrane is the sum of all three types of energy:

$$E_\mathrm{m} = E_\mathrm{l} + E_\mathrm{b} + E_\mathrm{s}. \tag{S14}$$

The elastic force acting on the membrane particles is then calculated using the principle of the virtual work as follows:

$$\mathbf{F}_i^\mathrm{elas} = -\frac{\partial E_\mathrm{m}}{\partial \mathbf{P}_i}, \tag{S15}$$

where $\mathbf{P}_i$ is the position vector of the ith node. The viscous force is given by

$$\mathbf{F}_i^\mathrm{vis} = -\gamma \mathbf{v}_{ij}, \tag{S16}$$

where γ is the viscosity coefficient of the cellular cytoskeleton, and v_{ij} is the relative displacement rate of the neighboring nodes i and j.

The nodal protrusive force ($\mathbf{F}_i^\mathrm{pro}$) is related to the local concentration of PIP_3 (P_3), and the nodal contractive force ($\mathbf{F}_i^\mathrm{con}$) is correlated with that of PIP_2 (P_2). The general forms of $\mathbf{F}_i^\mathrm{pro}$ and $\mathbf{F}_i^\mathrm{con}$ are given by

$$\mathbf{F}_i^\mathrm{pro} = \frac{\nu_\mathrm{pro} P_3}{2P_{3\mathrm{s}}}\left(l_i \hat{\mathbf{n}}_i + l_{i+1}\hat{\mathbf{n}}_{i+1}\right), \tag{S17}$$

$$\mathbf{F}_i^\mathrm{con} = \frac{-\nu_\mathrm{con} P_2}{2P_{2\mathrm{s}}}\left(l_i \hat{\mathbf{n}}_\mathbf{i} + l_{i+1}\hat{\mathbf{n}}_{i+1}\right), \tag{S18}$$

where ν_pro and ν_con are the protrusive and contractive force-concentration transfer factor, respectively, which are derived from the maximum active force generated by a cell, $P_{3\mathrm{s}}$ ($P_{2\mathrm{s}}$) is the saturation concentration of PIP_3 (PIP_2).

3.5 Chemotaxis index

The chemotaxis index (CI) of cells provided a measure of how well cell motion is directed toward an exogenous source. The instantaneous CI for cell i at time t is defined as

$$\mathrm{CI}(t, i) = \frac{\mathbf{v}_i \cdot \mathrm{r_i}}{|\mathbf{v}_i| \cdot |\mathrm{r_i}|}, \tag{S19}$$

Table S1. Physical and kinetic parameters of the signaling module

Parameter	Description	Value	Sources
D_s, D_c,D_m	Diffusion coefficients of extracellular, cytosolic, and membrane domains	100 (50), 10 (5-100), 1 $\mu\mathrm{m}^2 \cdot \mathrm{s}^{-1}$	[22]
k_d	Decay rate of chemoattractant	1 (0.5) s^{-1}	Estimated
k_p	Production rate of chemoattractant	10 $\mathrm{nm} \cdot \mathrm{s}^{-1}$	Estimated
GR	Total number of GPCRs	80000	[24, 26, 8]
δ_A, δ_I	Activator and inhibitor diminution rates	0.2, 0.2 s^{-1}	[11]
k_AI	Activator and inhibitor inactivation rate	10^2 (10^1 to $10^{2.6}$) $\mu\mathrm{m}/(\mathrm{s} \cdot \mathrm{molecule})$	[11]
k_s	Production rate of activator and inhibitor	1 s^{-1}	[11]
k_I	Association rate of inhibitor	3 $\mathrm{nM}^{-1} \cdot \mathrm{s}^{-1}$	[11]
R_tot, ρ_tot	Total levels of Rac, RhoA	7.5, 3 μM	[17, 15, 14]
R_b, ρ_b	Typical basal levels of active Rac, RhoA	3, 1.25 μM	[15, 14]
δ_R, δ_ρ	Basal decay rates of active Rac, RhoA	1, 1 s^{-1}	[14, 30]
I_R, I_ρ	Basal activation rates of Rac, RhoA	0.4, 0.4 s^{-1}	[25]
τ	Specific GEF-dependent RhoA activation rates	3 s^{-1}	Estimated
γ	RhoA-dependent Rac inactivation rate	1 $\mu\mathrm{m}(\mathrm{s} \cdot \mathrm{molecule})^{-1}$	[20]
P_2b, P_3b	Basal levels of PIP_2 and PIP_3	10, 0.45 μM	[14, 12]
$K_\mathrm{M}^\mathrm{PI3K}$, $K_\mathrm{M}^\mathrm{PTEN}$	PIP_2 and PIP_3 levels for half-max PI3K and PTEN feedback	8, 8 μM	Estimated
$k_\mathrm{cat}^\mathrm{PI3K}$, $k_\mathrm{cat}^\mathrm{PTEN}$	Enzymatic rates of PI3K and PTEN	8 (6-10), 8 (6-10) s^{-1}	[22]
k_slow, k_fast	Slow and fast release constants of FilGAP	0.1, 4 s^{-1}	[10]
β_1, β_2	Threshold angle of FLNa crosslink for slowly and fast releasing FilGAP	5°, 15°	Estimated

Table S2. Physical and kinetic parameters of the cell mechanics module

Parameter	Description	Value	Sources
d_N	diameter of the cell	10 μm	[29]
K_b	spring constant for bending	50 $\mathrm{pN} \cdot \mu\mathrm{m}$	[28, 6]
K_l	spring constant for stretching	5×10^5 $\mathrm{pN} \cdot \mu\mathrm{m}$	[28, 29]
K_s	penalty coefficient	5×10^5 $\mathrm{pN} \cdot \mu\mathrm{m}$	Estimated
γ	viscosity coefficient	0.5 $\mathrm{Pa} \cdot \mathrm{s}$	[28]
ν_pro, ν_con	protrusion and contraction coefficient	4, 3.2 pN/μm	Estimated

where $\mathbf{r}_i$ is the unit direction vector from cell i to the source point, v_i is the unit direction vector of the velocity of cell i. A CI value of 1 means that a cell is moving directly toward the source and thus fully responds to the information, whereas a CI value of 0 means motion perpendicular to the direction of the source and thus lacks the information about the micropipette position.

4 MODEL PARAMETERS

Model parameters are within the range of values previously measured and used in former models in the literature (see references in Table S1-S2). Parameters that are not well established were evaluated in the robustness analysis here as well as in our previous work [3].

4.1 Parameters for cell mechanics

The spring constants for cell bending (K_b) and stretching (K_l) were adopted from [28] upon the measurements of aspirated cells using a micropipette aspiration technique [29]. The penalty coefficient

was set to be at least one order of magnitude larger than K_l based on numerical rather than physical considerations. The force generated by a single actin filament can be estimated by the ratchet theory [21], which is a few tenths of the $\mathrm{pN}/\mu\mathrm{m}$. Considering that the density of F-actin at the leading edge is on the order of 10^6, the protrusive force density at the cell front can be estimated on the order of hundreds of pN. The total amount of contractive force can be experimentally derived from the traction force measurement [16], which is on the order of tens of nN.

4.2 Parameters for initial signaling processing

D_s and D_m are diffusive coefficients for chemoattractants and membrane-bound molecules (*i.e.*, activator and PIs), respectively. It is widely accepted that $D_s > 100\ \mu\mathrm{m}^2/\mathrm{s}$, and D_m is within the range of 0-1 $\mu\mathrm{m}^2/\mathrm{s}$ [22].

Since the guidance radius of chemoattractant L is on the order of hundreds $\mu\mathrm{m}$ and is generally not seen for distances $> 500\ \mu\mathrm{m}$ *in vivo* [5], L was set to be = 100 $\mu\mathrm{m}$ here. Accordingly, the space constant, λ, is estimated to be 30 $\mu\mathrm{m}$. Considering that 95% of the molecules are localized within a distance of 3λ from the source [22], the degradation rate of the chemoattractant can thus be estimated as $k_d = 0.1$ s. Given that the concentration limit that does not prevent gradient detection is 0.01-100 nM, C_b was set to 10 nM, a reasonable bottom bound of the concentration. Substituting the above parameters (with $f = 2\%$, a minimum percentage change detectable by a chemotactic cell) into Eq. S4, we may estimate $k_p = 10$ nM/s.

4.3 Parameters for cytoskeleton remodeling

Estimations of self-delay rates (δ_R, δ_ρ), and basal activation rates (I_R, I_ρ) for small Rho GTPases were based on the steady-state concentrations of their active forms, first achieved by [9]. and are involved in the bidirectional molecular transport module, which together determine the strength of Rho GTPase-PI feedback loops. A robustness analysis for two parameters was presented in our earlier work [3]. Briefly, only at the intermediate range of parameter combination (*i.e.*, 6 molecules/s $< k_{\mathrm{cat}}^{\mathrm{PI3K}} <$ 10 molecules/s) does the cell continually develop spontaneous motility.

4.4 Parameters in the mechanical-sensing module

Parameterizing the release rate of FilGAP upon stretching was performed by [10] in a minimally reconstituted system [2]. Accordingly, the experimentally derived, time-dependent fluorescence decay rate can be fitted with a two-exponential mixture model [10]. The control of the cytosolic concentration of FilGAP is also affected by the number of lamellipodial nodes (N). We thus carried out a robustness analysis on N (Figure S2), ensuring that the temporal evolution of [FilGAP] was not sensitive to N values.

5 NUMERICAL SIMULATION

The backbone of numerical simulations in our model is an iterative loop. Each time step occurs as follows:

1. At the beginning of the $(n + 1)$th time step, the system has already reached mechanical equilibrium. The FDM and MC steps are implanted to derive the concentration fields for both membrane-bound molecules and effector molecules.
2. The protrusive and contractive forces on membrane nodes are updated based on the concentration fields of PIP_3 and PIP_2, respectively.
3. The cell membrane is driven and deformed by membrane nodal forces.

4. The deformation of the actin network is calculated with the deformed cell membrane, providing the first boundary condition.
5. Once the geometry of the cell is updated, the cytosolic concentration of FilGAP is updated with the bandpass mechanism, and a new time step then begins.

5.1 One dimensional finite differences method

As the molecular diffusion on the membrane (circle) of 2D cell is one-dimensional, we use 1D finite difference to solve the diffusion equation on the cell membrane [28] according to Fick's laws. The 1D diffusion flux J of the molecule from node i to $i+1$ on the membrane,

$$J_i = -D\left(\frac{S_{i+1}/L_{i+1} - S_i/L_i}{\ell_i}\right) \tag{S20}$$

where the subscript i indicates the ith node on the cell membrane, ℓ_i is the edge length between node i and $i+1$, $L_i = (\ell_i + \ell_{i-1})/2$, D is the diffusivity on the membrane. Therefore, in one simulation time step Δt, the change of molecular concentration caused by diffusion at the ith node is

$$\Delta S_i = (J_{i-1} - J_i)\Delta t \tag{S21}$$

6 MODEL ASSUMPTIONS

Several simplifying assumptions are made in formulating the model, and the major assumptions are specified below.

1. In our model, we introduce a simple balanced-inactivation (BI) mechanism [11] for sensing chemoattractant stimuli. This parsimonious mechanism affords a natural role for achieving a switch-like distribution pattern of G_α and $G_{\beta\gamma}$. Other candidate directional sensing mechanisms involve the local modulation of chemoattractants [13] or a phase separation mechanism [4], although they are far more complex in mathematics.
2. The model assumes that the generation of protrusive and contractive forces is in accordance with PIP_3 and PIP_2, respectively. The relationship between PIP_3 regulation and protrusive force generation is abundant, as PIP_3 may provide membrane binding sites for actin binding proteins, such as Arp2/3 [1]. Moreover, fluorescent imaging data suggest that PTEN contains an N-terminal PIP_2 binding motif, and its deletion completely redistributes the enzyme into the cytosol [7, 27]. Since PTEN is an upstream signal transducer for myosin II localization, PIP_2 may regulate contractive force *via* myosin-II with the help of PTEN [23].
3. We assume that in the bidirectional molecular transport mechanism, the feedback loops between Rho GTPase and PIs are mediated *via* local recruitment and activation of PI3K and PTEN. However, experimental evidence indicates that PIP_3 also enhances Rac activity by promoting the localized activation of RacGEFs [1]. In earlier modeling works [14], such a feedback effect is included in PIP_3 concentration-dependent rate constants.
4. The translocation behavior of FilGAP between the cytosol and the membrane is not explicitly considered in our model. FilGAP contains an N-terminal PH (pleckstrin homology) domain, a GAP activity region, a spacer, and a C-terminal CC (coiled-coil) domain that mediates protein dimerization [19, 18]. It has been well established that the FilGAP C-terminal FLNa-binding site binds to 23 of 24 Ig-like repeats comprising the FLNa dimeric subunit structure (IgFLNa23) [18]. On the other hand,

although not firmly established, FilGAP's PH domain binds most strongly to PIP_3 after release from FLNa [18].

5. The model simply assumes FilGAP as the mediator for the antagonistic effect between Rac and RhoA. The evidence for Rac inactivation induced by FilGAP is sufficient. FilGAP can be phosphorylated by ROCK, a downstream effector of RhoA, and this phosphorylation stimulates its RacGAP activity, thereby antagonizing Rac signaling [19]. However, the role of FilGAP in RhoA is indirect. Rac inhibits RhoA through its activation of p190RhoGAP and inactivates the Rho exchange factor NET1 [18].

REFERENCES

[1]Pascale G Charest and Richard A Firtel. Feedback signaling controls leading-edge formation during chemotaxis. *Current Opinion in Genetics & Development*, 16(4):339–347, 2006.

[2]Allen Ehrlicher, Fumihiko Nakamura, John H Hartwig, David A Weitz, and Thomas P Stossel. Mechanical strain in actin networks regulates filgap and integrin binding to filamin a. *Nature*, 478(7368):260–263, 2011.

[3]Shiliang Feng, Lüwen Zhou, Yan Zhang, Shouqin Lü, and Mian Long. Mechanochemical modeling of neutrophil migration based on four signaling layers, integrin dynamics, and substrate stiffness. *Biomechanics and Modeling in Mechanobiology*, 17(6):1611–1630, 2018.

[4]Andrea Antonio Gamba, Antonio De Candia, Stefano Di Talia, Antonio Coniglio, Federico Bussolino, and Guido Serini. Diffusion-limited phase separation in eukaryotic chemotaxis. *Proceedings of the National Academy of Sciences of the United States of America*, 102(47):16927–16932, 2005.

[5]Geoffrey J Goodhill. Diffusion in axon guidance. *European Journal of Neuroscience*, 9(7):1414–1421, 1997.

[6]Marc Herant, Volkmar Heinrich, and Micah Dembo. Mechanics of neutrophil phagocytosis: behavior of the cortical tension. *Journal of cell science*, 118(9):1789–1797, 2005.

[7]Miho Iijima, Yi Elaine Huang, Hongbo R Luo, Francisca Vazquez, and Peter N Devreotes. Novel mechanism of pten regulation by its phosphatidylinositol 4,5-bisphosphate binding motif is critical for chemotaxis. *Journal of Biological Chemistry*, 279(16):16606–16613, 2004.

[8]Petrus M Janssens and PJ Van Haastert. Molecular basis of transmembrane signal transduction in dictyostelium discoideum. *Microbiological reviews*, 51(4):396, 1987.

[9]Alexandra Jilkine, Athanasius F M Maree, and Leah Edelsteinkeshet. Mathematical model for spatial segregation of the rho-family gtpases based on inhibitory crosstalk. *Bulletin of Mathematical Biology*, 69(6):1943–1978, 2007.

[10]John Kang, Kathleen M Puskar, Allen Ehrlicher, Philip R Leduc, and Russell Schwartz. Structurally governed cell mechanotransduction through multiscale modeling. *Scientific Reports*, 5(1):8622–8622, 2015.

[11]Herbert Levine, David A Kessler, and Wouterjan Rappel. Directional sensing in eukaryotic chemotaxis: A balanced inactivation model. *Proceedings of the National Academy of Sciences of the United States of America*, 103(26):9761–9766, 2006.

[12]Lan Ma, Chris Janetopoulos, Liu Yang, Peter N Devreotes, and Pablo A Iglesias. Two complementary, local excitation, global inhibition mechanisms acting in parallel can explain the chemoattractant-induced regulation of pi (3, 4, 5) p3 response in dictyostelium cells. *Biophysical journal*, 87(6):3764–3774, 2004.

[13]J A Mackenzie, M Nolan, and Robert H Insall. Local modulation of chemoattractant concentrations by single cells: dissection using a bulk-surface computational model. *Interface Focus*, 6(5):20160036–20160036, 2016.

[14]Athanasius FM Marée, Verônica A Grieneisen, and Leah Edelstein-Keshet. How cells integrate complex stimuli: the effect of feedback from phosphoinositides and cell shape on cell polarization and motility. *PLoS computational biology*, 8(3):e1002402, 2012.

[15]Athanasius FM Marée, Alexandra Jilkine, Adriana Dawes, Verônica A Grieneisen, and Leah Edelstein-Keshet. Polarization and movement of keratocytes: a multiscale modelling approach. *Bulletin of mathematical biology*, 68(5):1169–1211, 2006.

[16]Venkat Maruthamuthu, Benedikt Sabass, Ulrich S Schwarz, and Margaret L Gardel. Cell-ecm traction force modulates endogenous tension at cell–cell contacts. *Proceedings of the National Academy of Sciences of the United States of America*, 108(12):4708–4713, 2011.

[17]David Michaelson, Joseph Silletti, Gretchen Murphy, Peter D'Eustachio, Mark Rush, and Mark R Philips. Differential localization of rho gtpases in live cells: regulation by hypervariable regions and rhogdi binding. *The Journal of cell biology*, 152(1):111–126, 2001.

[18]Fumihiko Nakamura. Filgap and its close relatives: a mediator of rho-rac antagonism that regulates cell morphology and migration. *Biochemical Journal*, 453(1):17–25, 2013.

[19]Yasutaka Ohta, John H Hartwig, and Thomas P Stossel. Filgap, a rho- and rock-regulated gap for rac binds filamin a to control actin remodelling. *Nature Cell Biology*, 8(8):803–814, 2006.

[20]Mikiya Otsuji, Shuji Ishihara, Kozo Kaibuchi, Atsushi Mochizuki, Shinya Kuroda, et al. A mass conserved reaction–diffusion system captures properties of cell polarity. *PLoS computational biology*, 3(6):e108, 2007.

[21]Thomas D Pollard and Gary G Borisy. Cellular motility driven by assembly and disassembly of actin filaments. *Cell*, 112(4):453–465, 2003.

[22]Marten Postma and Peter J M Van Haastert. A diffusion-translocation model for gradient sensing by chemotactic cells. *Biophysical Journal*, 81(3):1314–1323, 2001.

[23]Md Kamruzzaman Pramanik, Miho Iijima, Yoshiaki Iwadate, and Shigehiko Yumura. Pten is a mechanosensing signal transducer for myosin ii localization in dictyostelium cells. *Genes to Cells*, 14(7):821–834, 2009.

[24]Wouter-Jan Rappel and Herbert Levine. Receptor noise and directional sensing in eukaryotic chemotaxis. *Physical review letters*, 100(22):228101, 2008.

[25]Yuichi Sakumura, Yuki Tsukada, Nobuhiko Yamamoto, and Shin Ishii. A molecular model for axon guidance based on cross talk between rho gtpases. *Biophysical journal*, 89(2):812–822, 2005.

[26]Masahiro Ueda, Yasushi Sako, Toshiki Tanaka, Peter Devreotes, and Toshio Yanagida. Single-molecule analysis of chemotactic signaling in dictyostelium cells. *Science*, 294(5543):864–867, 2001.

[27]Francisca Vazquez, Satomi Matsuoka, William R Sellers, Toshio Yanagida, Masahiro Ueda, and Peter N Devreotes. Tumor suppressor pten acts through dynamic interaction with the plasma membrane. *Proceedings of the National Academy of Sciences of the United States of America*, 103(10):3633–3638, 2006.

[28]Tenghu Wu and James J Feng. Modeling the mechanosensitivity of neutrophils passing through a narrow channel. *Biophysical Journal*, 109(11):2235–2245, 2015.

[29]Belinda Yap and Roger D Kamm. Mechanical deformation of neutrophils into narrow channels induces pseudopod projection and changes in biomechanical properties. *Journal of Applied Physiology*, 98(5):1930–1939, 2005.

[30]Baolin Zhang and Yi Zheng. Regulation of rhoa gtp hydrolysis by the gtpase-activating proteins p190, p50rhogap, bcr, and 3bp-1. *Biochemistry*, 37(15):5249–5257, 1998.
